# Assessment of the safety and probiotic properties of *Roseburia intestinalis*: A potential “Next Generation Probiotic”

**DOI:** 10.3389/fmicb.2022.973046

**Published:** 2022-09-08

**Authors:** Chao Zhang, Kejia Ma, Kai Nie, Minzi Deng, Weiwei Luo, Xing Wu, Yujun Huang, Xiaoyan Wang

**Affiliations:** ^1^Department of Gastroenterology, The Third Xiangya Hospital of Central South University, Changsha, China; ^2^Hunan Key Laboratory of Nonresolving Inflammation and Cancer, Cancer Research Institute, Central South University, Changsha, China

**Keywords:** probiotics, *Roseburia intestinalis*, safety, genome mining, oral toxicity, cytotoxicity, intestinal colonization

## Abstract

*Roseburia intestinalis* is an anaerobic bacterium that produces butyric acid and belongs to the phylum Firmicutes. There is increasing evidence that this bacterium has positive effects on several diseases, including inflammatory bowel disease, atherosclerosis, alcoholic fatty liver, colorectal cancer, and metabolic syndrome, making it a potential “Next Generation Probiotic.” We investigated the genomic characteristics, probiotic properties, cytotoxicity, oral toxicity, colonization characteristics of the bacterium, and its effect on the gut microbiota. The genome contains few genes encoding virulence factors, three clustered regularly interspaced short palindromic repeat (CRISPR) sequences, two Cas genes, no toxic biogenic amine synthesis genes, and several essential amino acid and vitamin synthesis genes. Seven prophages and 41 genomic islands were predicted. In addition to a bacteriocin (Zoocin A), the bacterium encodes four metabolic gene clusters that synthesize short-chain fatty acids and 222 carbohydrate-active enzyme modules. This bacterium is sensitive to antibiotics specified by the European Food Safety Authority, does not exhibit hemolytic or gelatinase activity, and exhibits some acid resistance. *R. intestinalis* adheres to intestinal epithelial cells and inhibits the invasion of certain pathogens. *In vitro* experiments showed that the bacterium was not cytotoxic. *R. intestinalis* did not affect the diversity or abundance of the gut flora. Using the fluorescent labelling method, we discovered that *R. intestinalis* colonizes the cecum and mucus of the colon. An oral toxicity study did not reveal any obvious adverse effects. The lethal dose (LD)50 of *R. intestinalis* exceeded 1.9 × 10^9^ colony forming units (CFU)/kg, whereas the no observed adverse effect level (NOAEL) derived from this study was 1.32 × 10^9^ CFU/kg/day for 28 days. The current research shows that, *R. intestinalis* is a suitable next-generation probiotic considering its probiotic properties and safety.

## Introduction

Probiotics are defined as “live microorganisms that, when administered in adequate amounts, confer a health benefit on the host” (Salminen et al., 2021). The global market for probiotics is expected to reach $91.1 billion by 2026, up significantly from $61.1 billion in 2021.^1^ Currently, most probiotic strains are derived from a limited number of bacterial species. These include *Lactobacillus* and *Bifidobacterium* species. Large-scale applications of metagenomic sequencing and bacterial genome editing methods have greatly expanded the range of bacteria with potential health benefits, and these bacteria are termed “Next Generation Probiotics” (NGPs; O’Toole et al., 2017). The development of NGPs requires by preclinical research, safety testing, pharmacokinetics, pharmacodynamics, and Phase 1–3 clinical trials.

*Roseburia intestinalis* is an anaerobic bacterium that colonizes in the intestine and belongs to the phylum Firmicutes (Duncan et al., 2002; Nie et al., 2021). During colitis, *R. intestinalis* modulates the immune response and maintains tight junction integrity (Luo et al., 2019). Its flagellin regulates the long noncoding RNA HIF1A-AS2 (Quan et al., 2018), inhibits the activation of the NOD-, LRR- and pyrin domain-containing protein 3 (NLRP3) inflammasome (Wu et al., 2020), and stimulates the differentiation of regulatory T (Treg) cells to suppress colitis (Zhu et al., 2018). *R. intestinalis* inhibits intestinal inflammation by increasing the secretion of anti-inflammatory cytokines such as thymic stromal lymphopoietin (TSLP), transforming growth factor (TGF)-β, and interleukin (IL)-10 (Shen et al., 2018). Moreover, *R. intestinalis* alleviates colitis by affecting the brain-gut axis (Xu et al., 2021).

**Figure 1:**
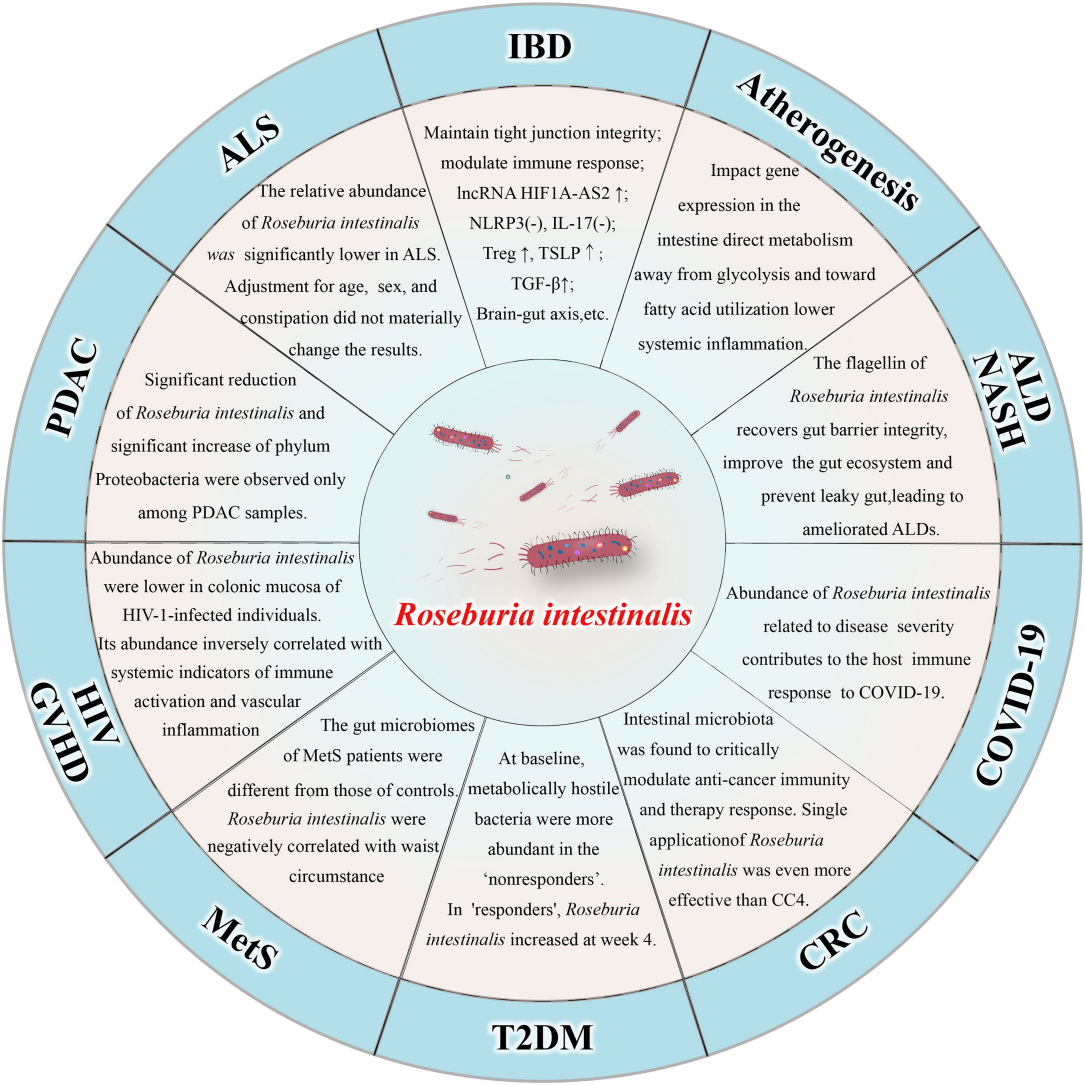
Relationship between *Roseburia intestinalis* and various diseases. IBD: inflammatory bowel disease; ALD: alcohol-related liver diseases; NASH: nonalcoholic steatohepatitis; COVID-19: coronavirus disease 2019; CRC: colorectal cancer; T2DM: type 2 diabetes mellitus; MetS: metabolic syndrome; HIV: human immunodeficiency virus; GVHD: Graft-versus-host disease; PDAC: pancreatic ductal adenocarcinoma; ALS: amyotrophic lateral sclerosis.

*Roseburia intestinalis* can also improve atherosclerosis (Kasahara et al., 2018; Liu et al., 2020) and alcohol-related liver diseases (Seo et al., 2020). There is evidence that the amount of *R. intestinalis* declines in patients with colorectal cancer (Montalban-Arques et al., 2021) and that treatment with the bacterium alone can prevent or even treat the disease (Liang et al., 2017). The abundance of *R. intestinalis* decreased in individuals suffering from amyotrophic lateral sclerosis (Nicholson et al., 2021), nonalcoholic steatohepatitis (Pan et al., 2021), pancreatic ductal adenocarcinoma (Zhou et al., 2021), and HIV infection (Dillon et al., 2017). *R. intestinalis* abundance was negatively correlated with waist circumference in patients with metabolic syndrome (Qin et al., 2021). Moreover, the bacterium has also been associated with graft-versus-host disease (Devaux et al., 2020) and insulin sensitivity in patients with type 2 diabetes (Lee et al., 2019). In fact, a recent study demonstrated *R. intestinalis* is associated with coronavirus disease 2019 (COVID-19), as the amount of the bacterium decreased significantly in severe patients (Xu et al., 2022). The relationship between *R. intestinalis* and various diseases is shown in Figure 1.

Currently, there are many guidelines related to probiotics, including those issued by the United States Food and Drug Administration (FDA), the World Gastroenterology Organization (WGO), the Product Safety Enforcement Forum of Europe (EU-PROSAFE; Vankerckhoven et al., 2008), and the International Scientific Association for Probiotics and Prebiotics (ISAPP; Hill et al., 2014). This study examined the safety and probiotic properties of *R. intestinalis* as well as its possible application in healthcare, in accordance with the above guidelines.

## Materials and methods

### Bacterial strains and culture conditions

*Roseburia intestinalis* L1-82 (DSMZ14610) was purchased from Deutsche Sammlung von Mikroorganismen und Zellkulturen GmbH (Braunschweig, Germany). *Bacteroides fragilis* (ATCC 25285) and *Bacteroides vulgatus* were obtained from Ningbo Mingzhou Biotechnology Co., Ltd. (Zhejiang, China). The above bacteria were cultured under anaerobic conditions at 37°C. An anaerobic culture environment was created using Atmosphere Generation Systems (AnaeroJar^™^ ASSEMBLY and AnaeroGen^™^, OXOID, Thermo Fisher Scientific). The culture medium (Miquel et al., 2015) was brain heart infusion (BHI) containing 0.5% yeast extract (OXOID), 1 mg/ml cellobiose (Macklin, China), 1 mg/ml maltose (Solarbio, China), 0.5 mg/ml cellobiose (Macklin, China), and 0.5 mg/ml cysteine (Solarbio, China). We established a standard curve to convert the absorbance values at 600 nm (OD600) to CFU values (Supplementary Figure S1). *Escherichia coli* ATCC 25922, *Staphylococcus aureus* ATCC 25923, *Salmonella typhimurium* CMCC 50115, *Bacillus subtilis* DSM 1088, and *Bacillus cereus* ATCC 11778 were used as controls. These strains were cultured in nutrient broth at 37°C in an aerobic environment.

### Genotypic characterization

#### Screening the *Roseburia intestinalis* genome for safety and probiotic-related traits

Genome data for *R. intestinalis* were obtained from the National Center for Biotechnology Information (NCBI) database (GCF_900537995.1). The Comprehensive Antibiotic Resistance Database (CARD; Alcock et al., 2020) and Virulence Factor Database (VFDB; Liu et al., 2022) were used to predict virulence and antibiotic resistance genes. Genes related to biogenic amines, essential amino acids, and vitamin synthesis were identified in the bacterial genome using the Rapid Annotations using Subsystems Technology (RAST) server (Overbeek et al., 2014). The Carbohydrate-Active enZYmes (CAZy; Drula et al., 2021) database was used to analyze the carbohydrate metabolism ability of *R. intestinalis*.

#### Prediction of the CRISPR-Cas system, prophages, bacteriocin, genomic islands, and primary metabolic gene clusters

CRISPRCasFinder was used to identify the CRISPR loci and associated Cas genes (Couvin et al., 2018). PHAge Search Tool Enhanced Release (PHASTER; Arndt et al., 2016) was used to identify and characterize the prophages within the genome. The bacteriocin operons were identified and visualized using BAGEL4 (van Heel et al., 2018). IslandViewer (Bertelli et al., 2017) was used to predict genomic islands (GIs) in the genome. All the above predictions were made using default parameters. GutSMASH (Pascal Andreu et al., 2021) was used to identify and analyze specific gene clusters associated with primary metabolism and energy capture.

#### Comparative genomic analysis

Several bacterial strains considered potential probiotics or pathogens in inflammatory bowel disease (IBD) were selected, and their genomes were compared with that of *R. intestinalis*. This included strains of *R. intestinalis* and other species of the genus *Roseburia*. GenBank accession numbers for these strains are listed in Supplementary Table S1. Genes involved in acid or bile salt tolerance, intestinal adhesion colonization, and antioxidant activity were retrieved from a previous study (Hussein et al., 2020) and aligned using the NCBI Basic Local Alignment Search Tool (BLAST) tools. We aligned the query genome with that of *R. intestinalis* using the BLAST Ring Image Generator (BRIG; Alikhan et al., 2011).

### Phenotypic safety assessment of probiotic characteristics

#### Acid tolerance

Acid tolerance was tested as previously described (Anandharaj et al., 2015). The bacterium was grown anaerobically at 37°C for 24 h, centrifuged at 4000 rpm for 10 min, and the suspension was washed twice with phosphate-buffered saline (PBS). We used 1 M HCL to adjust the pH of the medium in order to simulate the acidic environment of the stomach. *R. intestinalis* was added to the medium at varying pH values and cultured at 37°C for 24 h. Viable cells were counted on BHI agar plates, and the results were expressed as log_10_ colony-forming units (CFU)/ml.

#### Cell surface hydrophobicity

CSH was measured as described previously (Sharma et al., 2019). *R. intestinalis* in logarithmic growth phase was washed twice and resuspended in PBS, with the density adjusted to between 0.8 and 1.0. The n-hexadecane and bacterial solution were mixed at a ratio of 1:5 (v:v), and the mixture was vortexed for 2 min. The mixture was kept at room temperature for 45 min, and the aqueous phase was used to determine the OD600. CSH was calculated using the following formula:

CSH (%) = $\frac{A0-A}{A0}$ × 100.

A_0_ and A represent the OD600 values measured before and after mixing with n-hexadecane, respectively.

#### Auto-aggregation and co-aggregation

Auto-aggregation ability was identified based on the method described by Reuben et al. (2020), with slight modifications. The bacterium was rinsed twice and resuspended in PBS, and the OD600 was adjusted to 1.0 (A0). The bacterial suspension was incubated at 37°C for 2–24 h, and the OD600 of the supernatant was measured. Auto-aggregation capacity was calculated using to the following formula:

Auto-aggregation (%) = (1 - $\frac{At}{A0}$ ) × 100.

Co-aggregation ability was examined using a previously described method (Solieri et al., 2014). Equal volumes of *R. intestinalis* and pathogenic bacterial suspensions were mixed and vortexed for 10 s. A suspension of each bacterium alone was used as a control. A series of OD600 measurements were taken at different time points. The co-aggregation was calculated as follows:

Co-aggregation (%) = [1 - $\frac{A\left(x+y\right)}{\left(\mathrm{Ax}+Ay\right)/2}$ ] × 100.

For each time point, Ax represents the OD600 of *R. intestinalis*, and Ay represents the absorbance of each pathogenic bacterium.

#### Bile salt tolerance

The bile salt tolerance test was performed as previously described (Hussein et al., 2020). *R. intestinalis* at 10^8^ CFU/ml was inoculated into BHI medium containing 0.3% porcine bile salts (Solarbio, China). To simulate human intestinal conditions, 200 μl of bacterial broth was seeded in a 96-well microplate and incubated in an anaerobic incubator at 37°C for 0–8 h. Bacterial growth was monitored hourly by measuring OD600.

#### Bacterial adhesion to epithelial intestinal cells

The ability to adhere to intestinal epithelial cells was evaluated in a manner similar to that reported by Dudík et al. (2020). Caco-2 and HT-29 cells were cultured in Roswell Park Memorial Institute (RPMI)-1,640 medium containing 10% fetal bovine serum (Procell, China), seeded in six-well plates, and the medium was changed every 48 h. The cells were cultured continuously for 21 days. Bacteria were washed and resuspended in PBS (OD600 = 1.0). The plate was placed in a cell incubator at 37°C for 1 h with 1 ml of bacterial solution added to each well. The plate was rinsed three times with PBS, and 1 ml of 0.05% trypsin was added to digest the cells. The digested cell suspension was serially diluted in PBS and colonies were counted on BHI agar plates.

#### Hemolysin activity

The hemolytic activity experiments were based on Sonakshi et al. (Rastogi et al., 2020). *R. intestinalis* was inoculated onto BHI agar containing 5% (v/v) sterile defibrillated sheep blood, and cultured at 37°C under anaerobic conditions for 72 h. A clear halo around the colony indicates β-hemolysis, a green halo indicates α-hemolysis, and no halo indicates γ-hemolysis. *S. aureus* ATCC 25923 was used as a positive control.

#### DNase activity

DNase activity was measured using a previously described method (Rastogi et al., 2020). *R. intestinalis* was inoculated onto DNase agar medium (Hopebio, China) for 72 h. A solution of 1 M HCl was then added to the plate and the colonies were observed after a few minutes to determine whether a clear area was present around them. The positive control bacteria were *S. aureus* ATCC 25923 and the negative control bacteria were *E. coli* ATCC 25922.

#### Gelatinase activity

Approximately 50 μl of bacterial suspension (10^9^ CFU/ml) was added to a gelatin biochemical detection tube (Hopebio, China) and incubated at 37°C for 48 h. The tube was placed in a refrigerator for 20 min, and tilted to observe whether the medium could solidify. If the medium is liquid, gelatin is hydrolyzed and gelatinase activity is positive. *Bacillus cereus* ATCC 11778 was used as a positive control and *E. coli* ATCC 25922 was used as a negative control.

#### Detection of biofilm formation

*Roseburia intestinalis* was resuspended in the BHI medium and adjusted to a concentration of 10^7^ CFU/ml. Next, 200 μl of the bacterial suspension was added to a 96-well plate, and the plate was incubated at 37°C for 72 h in an anaerobic jar. The wells were rinsed thrice with PBS. Then, 200 μl of methanol was added to each well, and the methanol was removed after fixing for 15 min. Subsequently, 200 μl of 1% crystal violet was added, and the wells were washed three times with PBS after 15 min. After air-drying for 30 min, 200 μl of 30% glacial acetic acid was added to the plate. The liquid in the well was transferred to a new plate and the OD550 was measured. A 30% glacial acetic acid solution was used as blank control. As controls, we selected two anaerobic (*Bacteroides fragilis* and *Bacteroides vulgatus*) and one aerobic probiotic (*B. subtilis*), in addition to three other pathogens (*E. coli*, *S. aureus*, *S. typhimurium*). Using the average OD (ODc) of the negative control group as a cut-off value, the bacteria were categorized into those without biofilm (OD ≤ ODc), weak (ODc < OD ≤ 2ODc), moderate (2 ODc < OD ≤ 4ODc) and strong (OD > 4ODc) biofilm formation (Cozzolino et al., 2020).

#### Antibiotic susceptibility

According to European Food Safety Authority, probiotics must be tested for resistance to the following 9 antibiotics: ampicillin, vancomycin, gentamicin, kanamycin, streptomycin, erythromycin, clindamycin, tetracycline, and chloramphenicol. The disk diffusion method (Rocha-Mendoza et al., 2020) was used to determine the susceptibility of *R. intestinalis* to 17 antibiotics. By measuring the zone of inhibition according to Clinical and Laboratory Standards Institute (CLSI) criteria (CLSI, 2016), we were able to determine the susceptibility of the bacteria to antibiotics.

#### Inhibition of pathogen internalization

Three different methods (Fan et al., 2019) were used to examine the ability of *R. intestinalis* to defend against pathogen invasion of epithelial cells. HT-29 cells infected with 1 × 10^7^ CFU/ml of *E. coli* (multiplicity of infection [MOI] = 100) and incubated for 2 h served as a control group.

For the competition test, 1 × 10^8^ CFU/ml of *R. intestinalis* and 1 × 10^7^ CFU/ml of *E. coli* were added simultaneously to the cells and incubated for 2 h. For the displacement test, cells were first incubated with 1 × 10^7^ CFU/ml of *E. coli* for 1 h, and then 1 × 10^8^ CFU/ml of *R. intestinalis* was added and incubated for 1 h. For the exclusion test, HT-29 cells were first pre-incubated with 1 × 10^8^ CFU/ml of *R. intestinalis* for 1 h, followed by the addition of 1 × 10^7^ CFU/ml of *E. coli* for 1 h at 37°C. All groups were washed with PBS and the medium was changed to RPMI-1640 containing 1% penicillin/streptomycin/gentamicin (Biosharp, China) and incubated for 2 h at 37°C. To count intracellular *E. coli*, the cells were dissociated with 0.05% trypsin and treated with 0.1% Triton X-100 for 8 min. Results are expressed as the percentage decrease in *E. coli* invasion into cells in the experimental group compared with the control group.

### Determination of cytotoxicity

#### Cell culture

Two human colorectal cancer cell lines, HT-29 and Caco-2, as well as the normal human colon epithelial cell line NCM460, were cultured at 37°C in a humidified environment containing 5% CO2 in RPMI-1640 medium containing 10% fetal bovine serum (Procell, China). After the cells had grown to 80–90% confluency, they were digested with 0.05% trypsin and seeded into appropriate cell culture plates according to the experimental procedure.

#### Cell viability assay

The effect of *R. intestinalis* on cell viability was evaluated using a cell counting kit (CCK-8 assay). *R. intestinalis* was co-cultured with the cells for 4 h at an MOI of 100. The bacteria-containing medium was replaced with medium supplemented with 1% penicillin/streptomycin/gentamicin, followed by incubation at 37°C for 2 h to eliminate all extracellular bacteria (Li et al., 2021). For further experiments, the medium was changed to normal RPMI-1640 medium and cell viability was evaluated at different time points. The control group consisted of cells treated with *E. coli*.

#### Activity of lactate dehydrogenase

*Roseburia intestinalis* (10^8^ CFU/ml) was co-cultured with the NCM460 cells at 37°C for 4 h. Lactate dehydrogenase (LDH) released by the damaged cells was measured using an LDH assay kit (Beyotime, China). The results were expressed as a percentage of LDH activity in each group relative to the positive control reagent provided by the kit (Hussein et al., 2020).

#### Calcein-propidium iodide staining in live and dead cell detection test

HT-29 cells were co-cultured with bacteria (10^8^ CFU/ml) for 4 h, followed by a change to media containing 1% penicillin/streptomycin/gentamicin, and cultured for two additional hours at 37°C. The tests were conducted according to the manufacturer’s instructions (Beyotime, China), and the relative fluorescence values (RFU) were compared between the experimental and control groups.

#### Edu cell proliferation test

HT-29 cells were cultured in 6-well plates. As soon as the cells reached 80% confluence, 100 μl of *R. intestinalis* or *E. coli* (10^8^ CFU/ml) was added to the plate, and 30 μM of DNA synthesis inhibitor (hydroxyurea, Beyotime) served as the positive control. An EdU Cell Proliferation Kit (Beyotime) was used to detect cell proliferation after 24 h. The FUJI-ImageJ software was used to quantify the percentage of EdU-positive cells (Lu et al., 2019).

### Spatial and temporal distribution of *Roseburia intestinalis* within the gastrointestinal tract

#### Carboxyfluorescein diacetate succinimidyl ester staining of *Roseburia intestinalis*

The bacteria were centrifuged at 12000 rpm for 5 min and washed thrice with PBS. An equal volume of 1× CFDA-SE staining solution (Beyotime) was added to the bacterial suspension, and the mixture was incubated for 30 min at 37°C in the dark. The samples were then washed thrice with PBS to remove the unbound dye. Fluorescence was observed using a fluorescence microscope at an excitation wavelength of 488 nm.

#### Intestinal distribution of *Roseburia intestinalis* in C57BL/6 J mice

To investigate the characteristics of *R. intestinalis* colonization of the GIT, we used a previously described method (Zhao et al., 2022). *R. intestinalis* was collected during the logarithmic growth phase, stained with CFDA-SE, and resuspended in PBS at a concentration of 1 × 10^10^ CFU/ml. The mice were then gavaged with 200 μl of bacterial solution, whereas the control group was administered PBS. Mice were euthanized 2 to 72 h after gavage, and 1 cm long sections of the duodenum, jejunum, ileum, cecum, colon, and rectum were removed. After thoroughly rinsing the intestinal tube with PBS, the particulate matter was filtered using a 40 μm cell strainer. Flow cytometry (Cytek Athena^TM^) was used to analyze the ratio of CFDA-SE labelled *R. intestinalis* in the intestine. Mice gavaged with non-fluorescently labelled *R. intestinalis* were used as blank controls.

For frozen sections, mice were euthanized 12 h after administration of *R. intestinalis*, cecal and colonic specimens were collected and washed in sterile PBS, embedded with Optimal cutting temperature (OCT) reagent (Sakura, Japan), and frozen sections were obtained immediately (Leica CM1950).

#### Cecal microbiota

To analyze the effect of *R. intestinalis* on the structure of the intestinal flora, mice were gavaged with *R. intestinalis* at a concentration of 1 × 10^9^ CFU/ml for 14 days. On days 7 and 14, mouse feces were harvested for 16S rRNA sequencing.

A MagPure Stool DNA kit (Magen, China) was used to extract DNA from the microbial communities. DNA was quantified using a Qubit Fluorometer with the Qubit dsDNA Kit (Invitrogen, United States), and quality was checked using a 1% agarose gel. Polymerase chain reaction (PCR) primers 341F (5’-ACTCCTACGGGAGGCAGCAG-3′) and 806R (5’-GGACTACHVGGGTWTCTAAT-3′) were used to amplify the variable regions V3–V4 of the bacterial 16S rRNA genes. The primers were tagged with Illumina adapter, pad, and linker sequences. PCR enrichment was performed using a 50 μl reaction containing 30 ng template, fusion PCR primer, and master mix. The PCR cycling conditions were 94°C for 3 min, 30 cycles of 94°C for 30 s, 56°C for 45 s, 72°C for 45 s, and 10 min at 72°C for extension. PCR products were purified using AmpureXP beads and eluted with elution buffer. The libraries were qualified using an Agilent 2,100 bioanalyzer (Agilent, United States). The validated libraries were sequenced on an Illumina MiSeq platform (BGI, Shenzhen, China) following the standard Illumina pipeline and 2 × 300 bp paired-end reads were generated.

### *In vivo* toxicology studies

#### Animals and tests organisms

Oral toxicity studies were performed using male C57BL/6 J mice (6–8 weeks) and all animal experiments were approved by the Central South University Animal Ethics Committee (XMSB-2022-0198). The animals were housed under standard conditions with alternating periods of light and dark (12 h each) at a temperature of 25 ± 2°C and with free access to food and water. The animals were acclimated for 7 days before the experiment. *R. intestinalis* was cultured in an anaerobic environment at 37°C for 24 h in the BHI medium. Bacteria were centrifuged for 10 min at 4000 rpm, rinsed, and resuspended in PBS.

#### Acute oral toxicity study

The acute oral toxicity test was conducted according to the method described by Anantha et al. (Metlakunta and Soman, 2020) and the Organisation for Economic Co-operation and Development (OECD) guideline No.423 with certain modifications. This study was conducted in a stepwise manner. As a first step, three mice (group A1) were gavaged with *R. intestinalis* at a dose of 1.9 × 10^9^ CFU/kg. It represents 133–1,330 times the empirical dose level of oral probiotics in humans (i.e., 108–10^9^ CFU or 1.43 × 106–1.43 × 10^7^ CFU/kg/day in a 70 kg individual; Steppe et al., 2014). In the following 48 h, there were no mortality, morbidity, or abnormal clinical signs. To confirm this, the same dosage was administered to another group (A2), while a control group of mice (A0) was administered PBS. The animals were monitored for 14 consecutive days for clinical manifestations, such as changes in the skin and fur, mucous membranes, respiration, and behavioral changes. The animals were euthanized at the end of the observation period, and the gross appearance of vital organs, organ weights, and histopathology were assessed. Blood was collected from the animals for biochemical and hematological analyses.

#### 28-day repeated dose toxicity study

According to OECD No.407, this part of the study evaluated the toxicity of *R. intestinalis* after repeated administration for 28 days to establish dose–response relationships and determine the NOAEL. Each group consisted of six mice, and the control group (S0) was administered PBS *via* gavage. The experimental groups were divided into three dose levels corresponding to 66–657 times (S1), 77–769 times (S2), and 92–923 times (S3) the empirical dose of oral probiotics in humans. The mice were monitored daily for changes in clinical signs, mortality, fur and skin, respiration, behavioral patterns, body weight, food intake, and water consumption. On the 29th day, the mice were euthanized and blood samples were collected for analysis of hematology and biochemical indicators. The gross appearance of vital organs, organ weights, and histopathology were assessed.

### Statistical analysis

Statistical analyses were performed using SPSS version 25.0. To ensure homogeneity of variance and normality of the data, the Kolmogorov–Smirnov test was used. The values are presented as the mean ± standard deviation for normally distributed data or as the median and interquartile range for data that were not normally distributed. When comparing two groups, *p* values were derived from the Student’s t-test for normally distributed data, and the Mann–Whitney was used for data with other distributions. For multi-group comparisons, *p* values were determined using one-way ANOVA. We considered *p* < 0.05 statistically significant in all comparisons.

## Results

### Genome annotation

**Figure 2:**
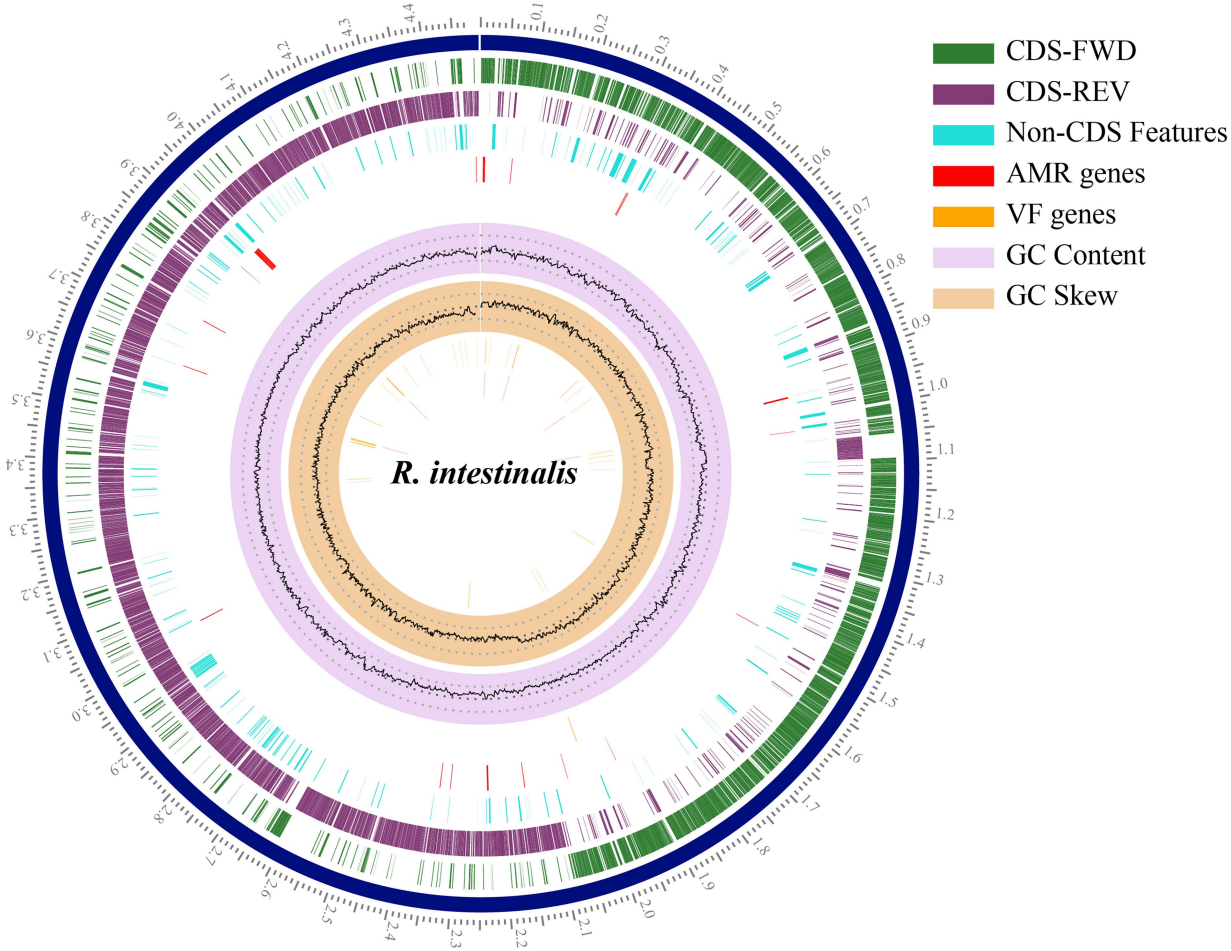
Circular genome map of *Roseburia intestinalis*. The outermost scales indicate the base location in Mbp. From the innermost circles: GC skew, GC content, virulence factor genes, AMR genes, Non-CDS features. The last two circles illustrate the coding sequence, the green circle represents the forward strand, and the purple circle represents the backward strand.

The RAST annotation results indicated that the genome contained 4,340 coding sequences and 81 RNA genes (Supplementary Figure S2). In total 252 subsystems were functionally annotated. Carbohydrates accounted for the largest number of genes (200), followed by amino acids and derivatives (192). Pathosystems Resource Integration Center (PATRIC; Davis et al., 2020) was used to generate a circular genome map (Figure 2).

### Safety and probiotic-related traits in *Roseburia intestinalis* genome

In *Roseburia intestinalis*, we detected three CRISPR sequences and two Cas genes. The CRISPR sequences were located at 1020388–1020441 bp, 1,222,931–1,223,608 bp, and 1,225,355–1,226,103 bp, while the Cas gene sites were located at 1015665–1019051 bp and 1,216,581–1,217,327 bp. The genome contained no genes involved in biogenic amine biosynthesis other than spermidine synthase (*speE*) and carboxynorspermidine decarboxylase (*nspC*). The genome contains essential amino acid synthesis genes (tryptophan, methionine, threonine, arginine, and cysteine), as well as vitamin synthesis genes (i.e., Thiamine, Riboflavin, Folate, Pantothenate, and Biotin; Table 1).

**Table 1:** Associated biosynthetic genes detected in *R. intestinalis* genome about essential amino acids or vitamins.

Category	Name	KO	Gene	Biosynthesis protein
Essential amino acid	Tryptophan	ko: K01695	trpA	tryptophan synthase alpha chain
		ko: K01696	trpB	tryptophan synthase beta chain
		ko: K01867	WARS	tryptophanyl-tRNA synthetase
	methionine	ko: K00789	metK	S-adenosylmethionine synthetase
		ko: K02071	metN	D-methionine transport system ATP-binding protein
		ko: K02072	metI	D-methionine transport system permease protein
		ko: K02073	metQ	D-methionine transport system substrate-binding protein
	threonine	ko: K01733	thrC	threonine synthase
		ko: K01620	ltaE	threonine aldolase
		ko: K04720	cobD	threonine-phosphate decarboxylase
	Arginine	ko: K01585	speA	arginine decarboxylase
	Cysteine	ko: K01738	cysK	cysteine synthase
		ko: K04487	iscS	cysteine desulfurase
Vitamins	Thiamine	ko: K00788	thiE	thiamine-phosphate pyrophosphorylase
		ko: K00949	thiN	thiamine pyrophosphokinase
	Riboflavin	ko: K00793	ribE	riboflavin synthase
	Folate	ko: K11754	folC	dihydrofolate synthase / folylpolyglutamate synthase
		ko: K00287	DHFR	dihydrofolate reductase
	Pantothenate	ko: K03525	coaX	type III pantothenate kinase
	Biotin	ko: K01012	bioB	biotin synthase

### Prophages, bacteriocins, metabolic gene clusters, carbohydrate-active enzymes, and genomic islands

**Figure 3:**
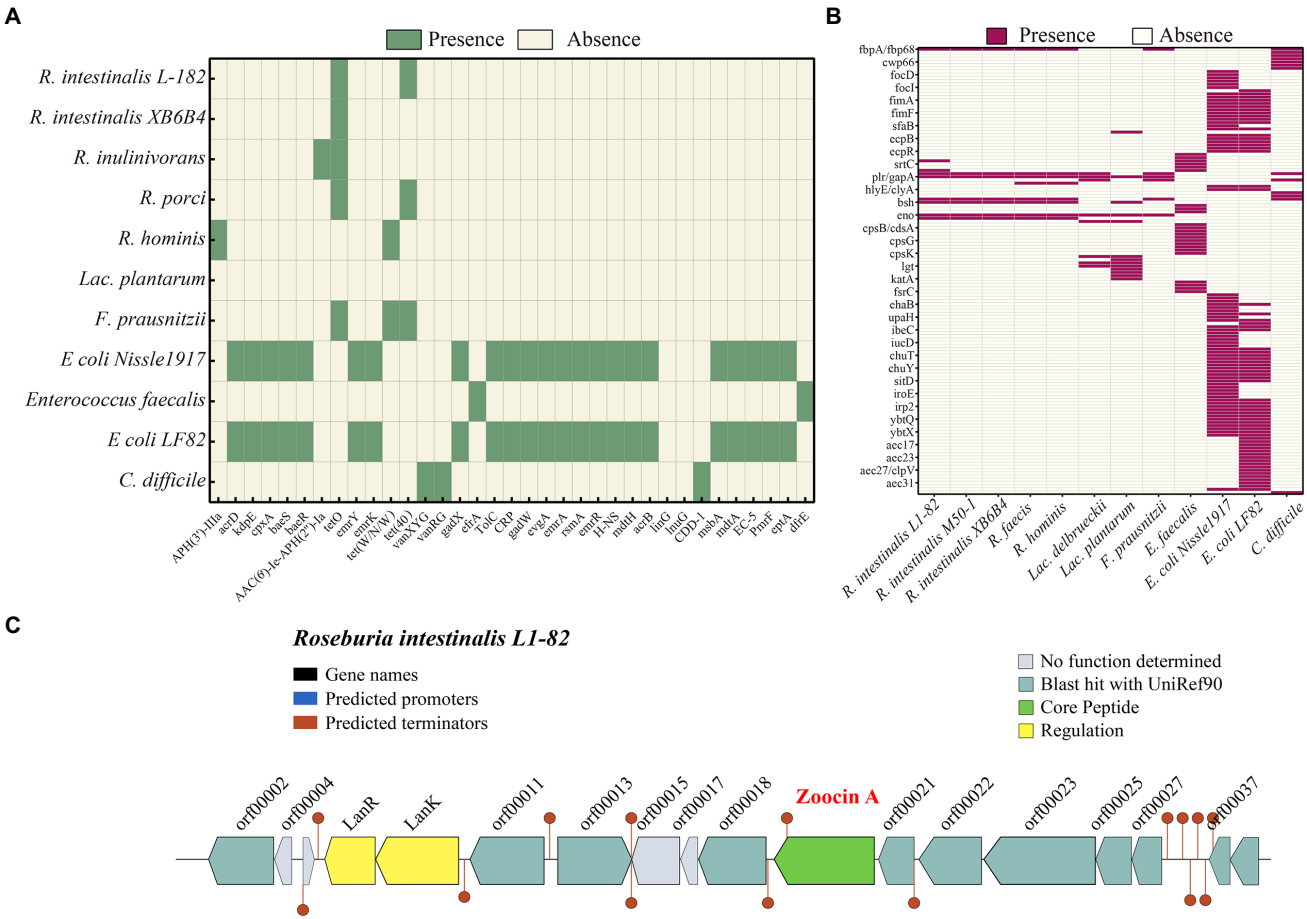
Heatmap showing the clustering of the compared *R. intestinalis* genomes based on the presence of antibiotic resistance genes **(A)** and virulence genes **(B)**. **(C)** Organization of bacteriocin cluster genes. orf00002, RNA pseudouridine synthase; orf00011, S-adenosylmethionine synthase; orf00013, Cyclic di-GMP phosphodiesterase; orf00018, UDP-N-acetylglucosamine 1-carboxyvinyltransferase; orf00021, Shikimate kinase; orf00022, Foldase protein PrsA; orf00023, Transcription-repair-coupling factor; orf00025, Peptidyl-tRNA hydrolase; orf00027, Bifunctional protein GlmU; orf00037, Putative septation protein SpoVG; orf00039, Glucose-1-phosphate adenylyltransferase.

PHASTER identified seven prophages within the genome, of which three are incomplete and four are questionable. The details of the prophages are provided in Supplementary Table S2. Using the BAGEL4 webserver, we detected that the bacterium harbored a bacteriocin cluster (Figure 3C) of Zoocin A class within contig 93.3 (3723994–3,744,393 bp). AOI consisted of RNA pseudouridine synthase, a transcriptional regulatory protein (LanR), a putative lantibiotic resistance two-component sensor kinase precursor (LanK), and multiple open reading frames (ORFs). Using gutSMASH, we discovered that the genome contained six metabolism-related gene clusters (Table 2). Four of these clusters are associated with short-chain fatty acid metabolism, including butyric acid synthesis.

**Table 2:** Identified primary metabolite regions by gutSMASH.

Region	Type	Class	From	To	Core biosynthetic genes	Similarity
1	porA	SCFA	54,788	78,257	porB,porC	
2	acetate to butyrate	SCFA	642,855	668,098	thlA, hbd,bcd, carD,carE	83%
3	Rnf complex,succinate to propionate	SCFA	2,259,537	2,304,034	mcp1,rsxB2,rsxA, rnfE,rsxG,rsxD, rsxC,gcdB,pccB	100%
4	Putrescine to spermidine	Aliphatic amine	3,070,869	3,094,310	nspC,speE	100%
5	Others HGD unassigned	Putative	3,145,614	3,169,501	hgdC	
6	Pyruvate to acetate-formate	SCFA	3,615,206	3,638,200	pflA,pflB	100%

The CAZy database identified 222 carbohydrate-active enzyme modules in the genome. A total of 136 glycoside hydrolase families, 48 glycosyltransferases, 14 carbohydrate esterase families, and 24 carbohydrate-binding module families were identified. Using IslandViewer, we combined two prediction algorithms, islandPath-DIMOB and SIGI-HMM, and identified 41 GIs (Supplementary Figure S3). In the predicted GIs, no annotated virulence, antibiotic resistance, or pathogenicity genes were identified.

### Comparative genomic analysis

The genome contains *tetO* and *Tet(40)*, which are associated with tetracycline resistance, whereas other *Roseburia* genera and other probiotic/pathogenic bacteria contain more antibiotic resistance genes (Figure 3A). According to VFDB, the *R. intestinalis* genome contains genes related to adhesion (*fbpA/fbp68*, *ebpC*, *efaA*, *plr/gapA*), and lipid and fatty acid metabolism (*panD*; Figure 3B). Compared to other probiotics (all belonging to Firmicutes), *R. intestinalis* contains more genes that encode cell adhesion-related proteins and genes related to gastrointestinal survival (Supplementary Figure S4). The genome contains seven genes related to antioxidant activity, suggesting a strong ability to survive in the host environment. Furthermore, we compared its genome with the genomes of six probiotics and found that *F. prausnitzii*, a probiotic used in the treatment of IBD (Machiels et al., 2014), possessed the highest degree of similarity (Supplementary Figure S5).

### Phenotypic safety assessment of probiotic characteristics

*R. intestinalis* was tested for its resistance to 17 antibiotics (Table 3). The bacterium was sensitive to most antibiotics, had intermediate resistance to streptomycin and kanamycin, and was only resistant to amikacin.

**Table 3:** Antibiotic susceptibility of selected antibiotics tested against *R. intestinalis.*

Antibiotic	ZOI (mm)	Antibiotic susceptibility	Antibiotic	ZOI (mm)	Antibiotic susceptibility
Amikacin	13.5 ± 0.41	R	Penicillin	46.1 ± 0.25	S
Ampicillin	19.6 ± 0.46	S	Gentamicin	17.6 ± 0.49	S
Erythromycin	48.4 ± 0.29	S	Tetracycline	27.7 ± 0.53	S
Kanamycin	14.1 ± 0.22	I	Cefuroxime	36.7 ± 2.05	S
Clindamycin	40.2 ± 0.54	S	Cefoperazone	39.7 ± 4.49	S
Chloramphenicol	38.9 ± 1.63	S	Ceftriaxone	40. 7 ± 3.09	S
Streptomycin	14.5 ± 0.45	I	Ceftazidime	21.3 ± 0.47	S
Minocycline	40.7 ± 1.69	S	Vancomycin	27.5 ± 0.33	S
Piperacillin	37.8 ± 0.85	S			

S: susceptible; I: intermediate; R: resistant; ZOI: Zone of growth inhibition.

**Figure 4:**
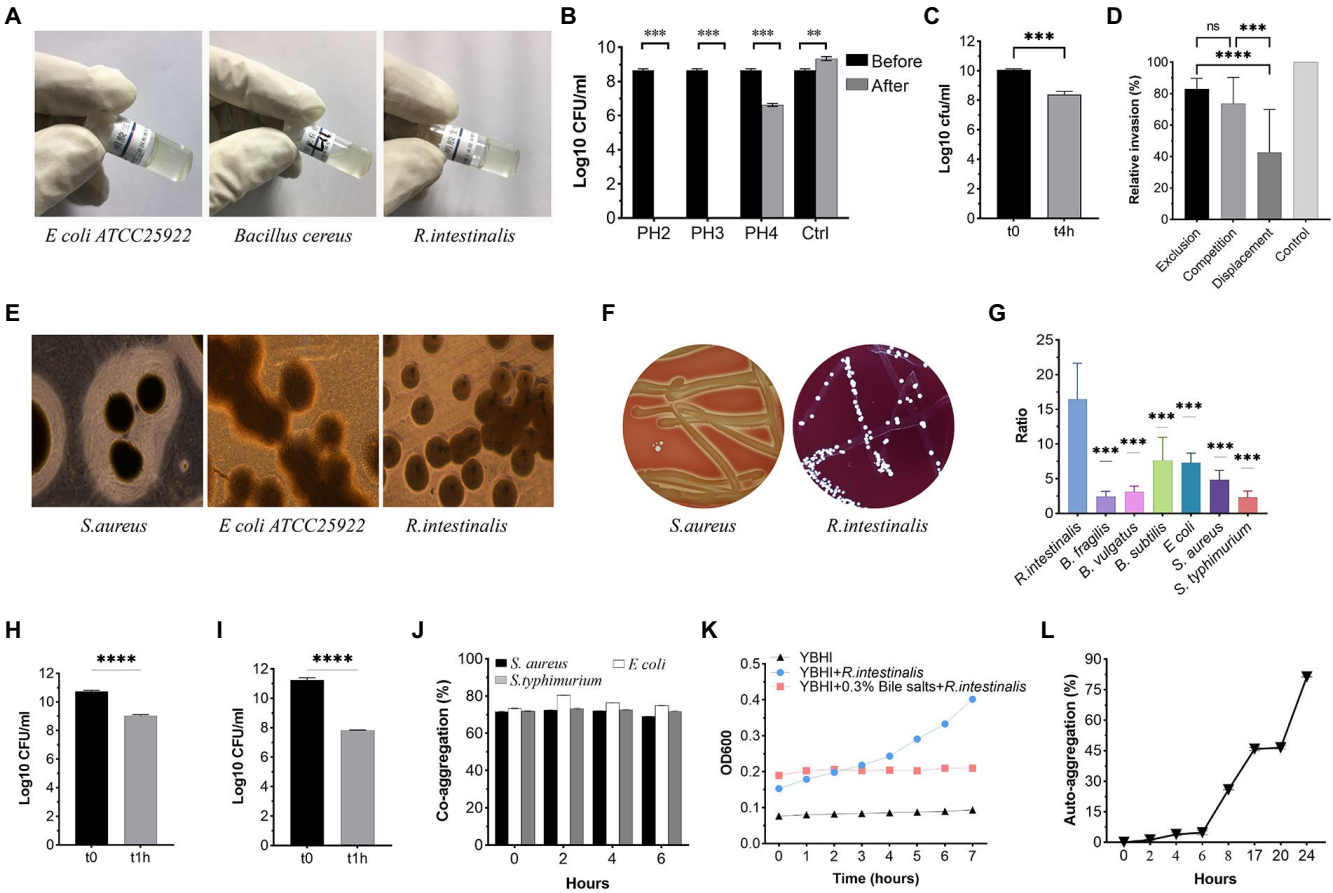
Phenotypic safety assessment of probiotic Characteristics. **(A)** Gelatinase activity of *R. intestinalis* compared to *Bacillus cereus* ATCC 14579 (positive control) and *E coli* ATCC 25922 (negative control). **(B)** Acid tolerance. **(C,K)** Bile salt and artificial intestinal fluid tolerance. **(D)** Inhibition of internalization of pathogens. **(E)** DNase activity. A clear zone around the colony represents DNase activity. **(F)** Hemolytic activities. **(G)** Biofilm formation. The y-axis represents the ratio of the OD550 of the bacteria to the ODc. **(H,I)** Adhesion of *R. intestinalis* on Caco-2 and HT-29 cells. **(J,L)** Co-aggregation and auto-aggregation abilities. * indicates *p* < 0.05, ns indicates no significance.

As shown in Figure 4, *R. intestinalis* exhibited no gelatinase, (Figure 4A) DNase (Figure 4E), or hemolytic activity. As a positive control, *S. aureus* ATCC 25923 exhibited β-hemolysis (Figure 4F). *R. intestinalis* survived in an environment with a pH of 4 (Figure 4B). The proliferation of bacteria was inhibited in the presence of 0.3% bile salt, whereas bacteria survived in the artificial intestinal fluid (Figures 4C,K). *R. intestinalis* was able to adhere effectively to HT-29 and Caco-2 cells (Figures 4H,I) and exhibited a hydrophobicity of 12.25 ± 1.01% in n-hexadecane. At 24 h, *R. intestinalis* had a maximum auto-aggregation ability of 80% (Figure 4L), and the co-aggregation experiment (Figure 4J) showed a strong co-aggregation ability (69–80%). Competition, displacement, and exclusion experiments showed that *R. intestinalis* inhibited the internalization of *E. coli* into epithelial cells (Figure 4D). *R. intestinalis* had a higher biofilm-forming ability than several probiotics and pathogens (Figure 4G). This may allow it to gain a competitive growth advantage and enhance its ability to adhere to the intestinal epithelium.

### Cytotoxicity

**Figure 5:**
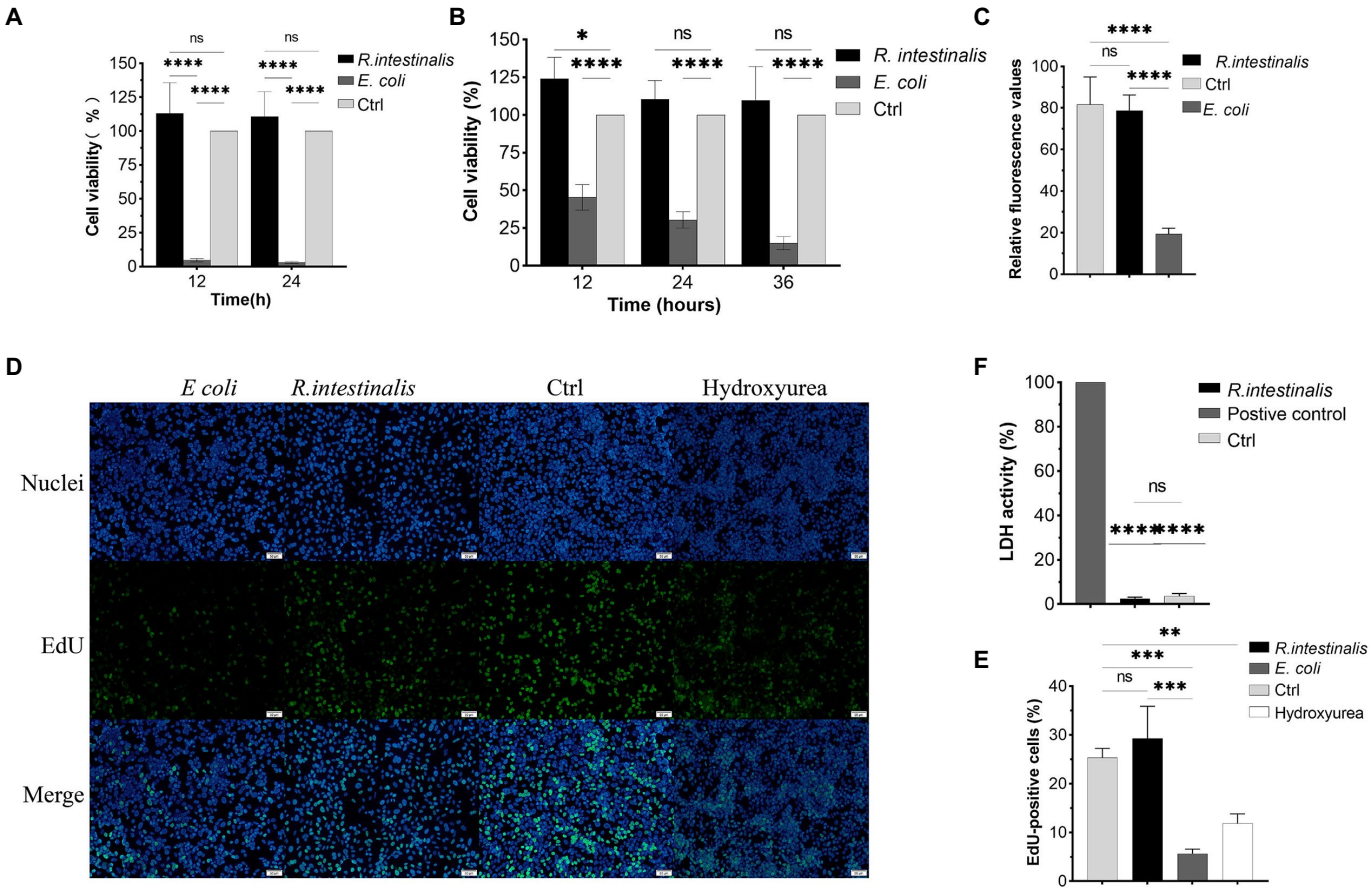
Cytotoxic effects of *R. intestinalis*. **(A,B)** Cytotoxic effects of *R. intestinalis* on HT-29 and NCM460 cells after 12–36 h of treatment. **(C)** Calcein-PI staining in live and dead cell detection test. **(D,E)** EdU cellular proliferation test. The hydroxyurea treatment group served as the positive control. **(F)** LDH activity test. * indicates *p* < 0.05, ns indicates no significance.

After treatment with *R. intestinalis*, there was no statistically significant difference in cell viability between the experimental and control groups (*p* > 0.05); however, cell viability was significantly reduced after treatment with *E. coli* (Figures 5A,B). As shown in Figure 5C, the RFU of the experimental group was not significantly different from that of the control group, whereas that of the *E. coli* treatment group was significantly different (*p* < 0.05). Compared to the positive control, there was no significant difference in LDH activity between the *R. intestinalis* treatment and control groups. This indicates that this probiotic bacterium did not cause notable cell damage (Figure 5F). The proportion of EdU-positive cells in the treatment group did not differ significantly from that in the control group but differed significantly from that in the positive control group (*p* < 0.05). This indicated that *R. intestinalis* treatment did not affect DNA replication during cellular proliferation (Figures 5D,E).

### Cecal microbiota

**Figure 6:**
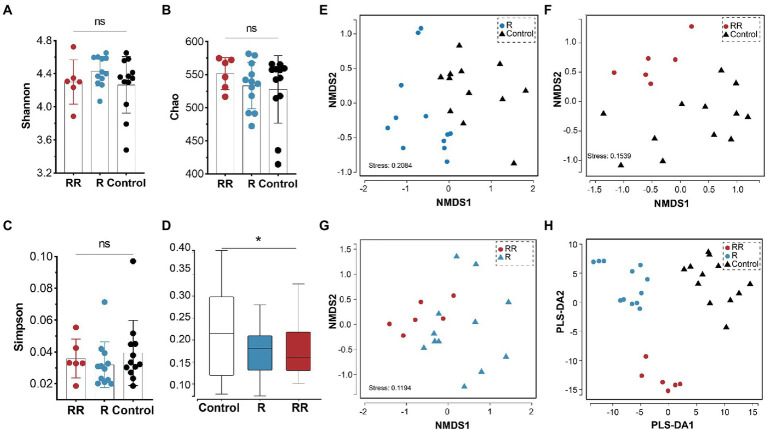
Cecal microbiota α-diversity and β-diversity following administration of *R. intestinalis* for 7 days (R group) or 14 days (RR group). **(A)** Shannon’s index. **(B)** Chao’s index. **(C)** Simpson’s index. **(D)** Weighted UniFrac distance of the cecal microbiota. **(E–G)** NMDS analysis among groups. **(H)** Partial least squares discrimination analysis (PLS-DA) analysis. * indicates *p* < 0.05, ns indicates no significance.

**Figure 7:**
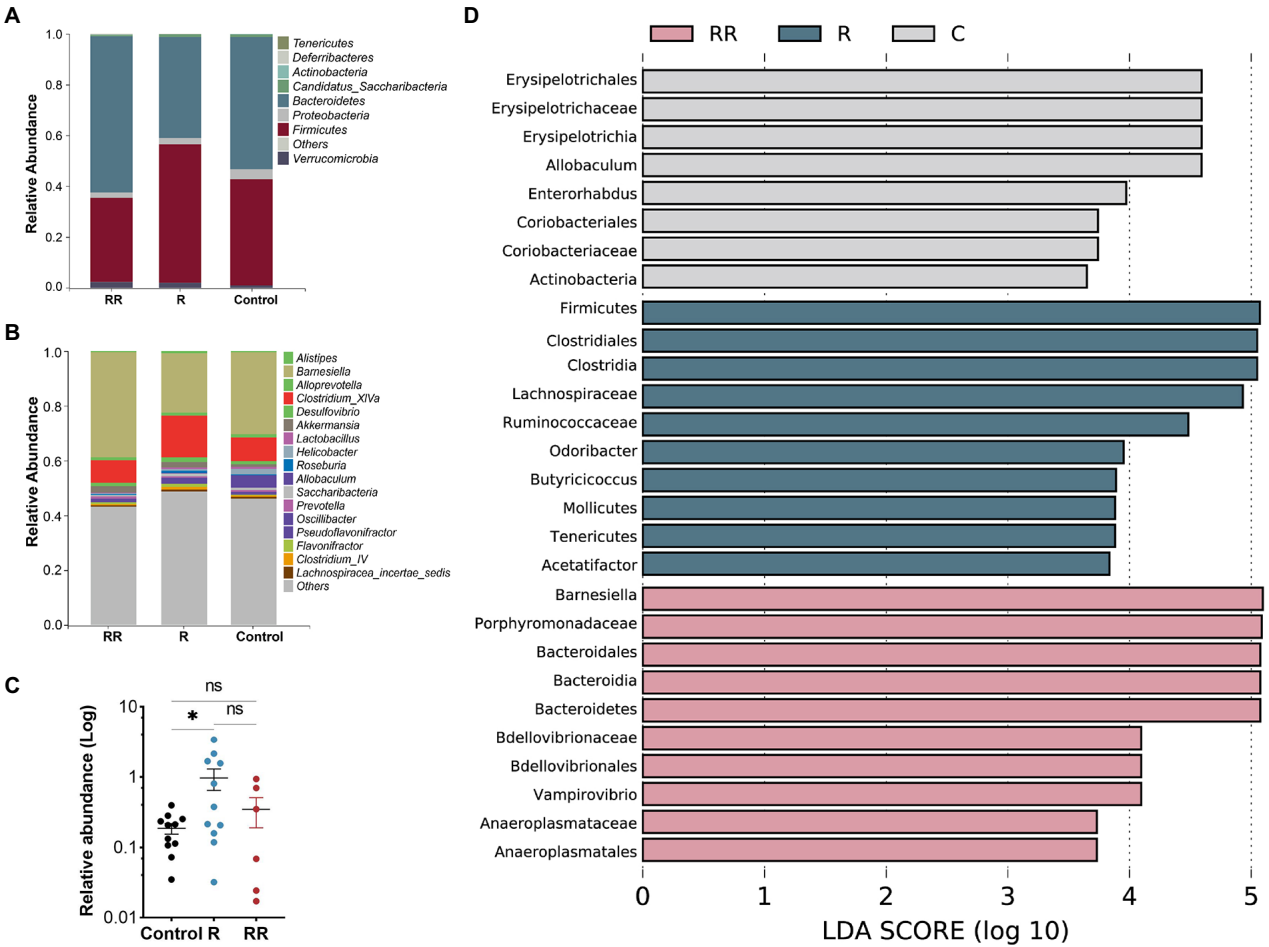
Classification of microflora at the phylum **(A)**, and genus **(B)** levels. **(C)** Relative abundance of *Roseburia intestinalis* among different groups. R: 7 days group; RR: 14 days group. **(D)** LEfSe analysis between different groups, displaying only genera with absolute values of linear discriminant analysis (LDA) scores greater than 2.0. * indicates *p* < 0.05, ns indicates no significance.

There was no statistically significant difference among the groups in any of the α-diversity indices (Figures 6A–C), indicating that oral administration of *R. intestinalis* did not modify gut microbiota diversity and abundance. Non-metric multidimensional scaling (NMDS) analysis using a weighted UniFrac analysis revealed distinct differences in microbiota structure (Figures 6D–H). We found that the highest relative abundance of *R. intestinalis* occurred 7 days after oral administration (Figures 7A-C). Species with core effects were analyzed using the LEfSe method. The core strains after 7 days of administration were *Firmicutes*, *Clostridiales*, *Clostridia*, *Lachnospiraceae* among others. After 14 days, the core species were *Barnesiella* and *Porphyromonadaceae* (Figure 7D). The PICRUSt2 tool based on the Kyoto Encyclopedia of Genes and Genomes (KEGG) database was used to predict pathway enrichment, and we observed differences in pathway enrichment after the administration of *R. intestinalis* (Supplementary Figure S6).

### Colonization characteristics of *Roseburia intestinalis* using fluorescent labelling

**Figure 8:**
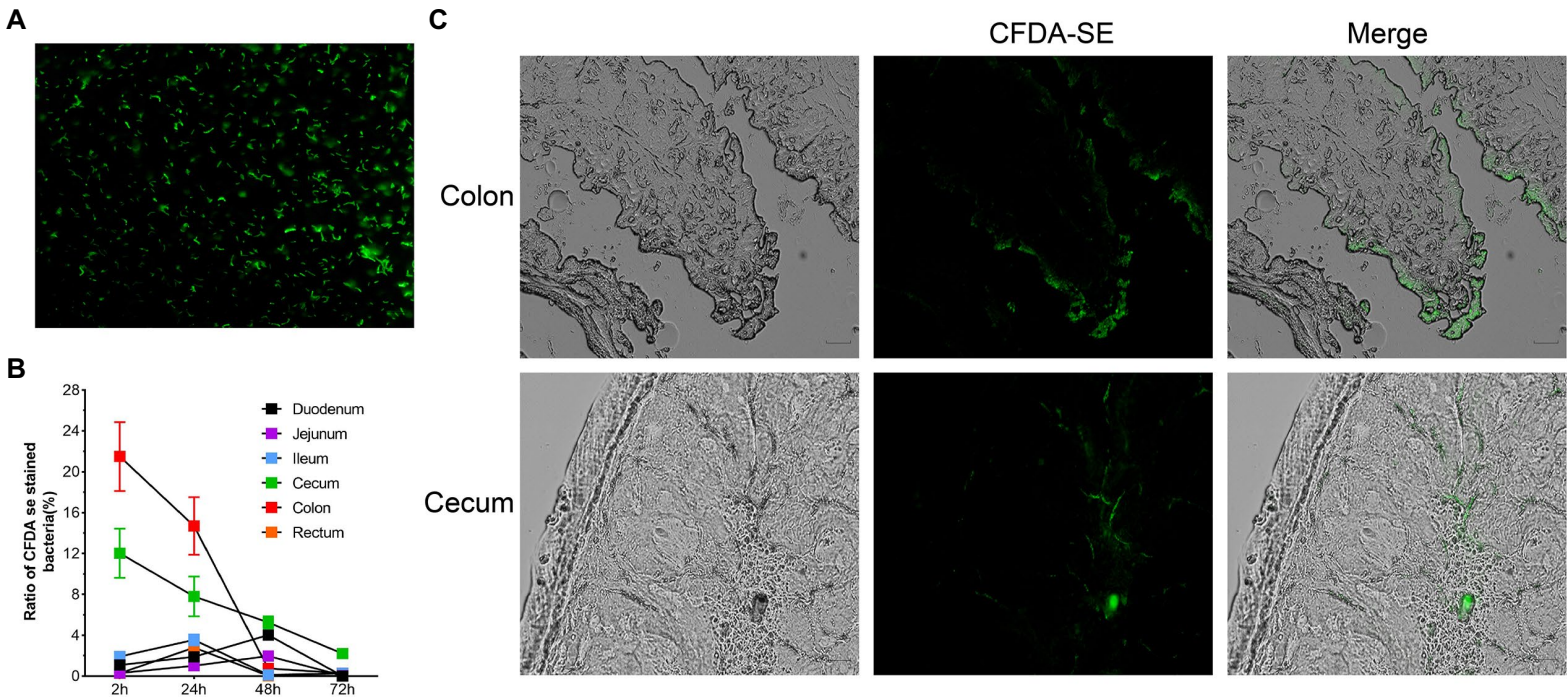
Using flow cytometry and frozen sections to characterize the colonization of *Roseburia intestinalis* in the GIT. **(A)** CFDA-SE labelled *R. intestinalis* investigated under fluorescence microscopy. **(B)** *R. intestinalis* fluorescence at different time points and in different areas of the intestine. **(C)** Frozen sections were used to observe the distribution of *R. intestinalis* in the colon and cecum.

Two hours after oral administration, fluorescently labelled bacteria (Figure 8A) were detected in all intestinal segments, with the highest concentration in the colon. After 48 h, the content of *R. intestinalis* in the cecum exceeded that in the colon. After 72 h, the bacterium primarily colonized the cecum and colon, whereas its distribution in the duodenum, jejunum, ileum, and rectum was scarce (Figure 8B; Supplementary Figure S7). The results of the frozen section demonstrated that the spatial colonization sites of *R. intestinalis* were primarily located in the cecum and colonic mucus layers (Figure 8C).

### Acute toxicity study

**Figure 9:**
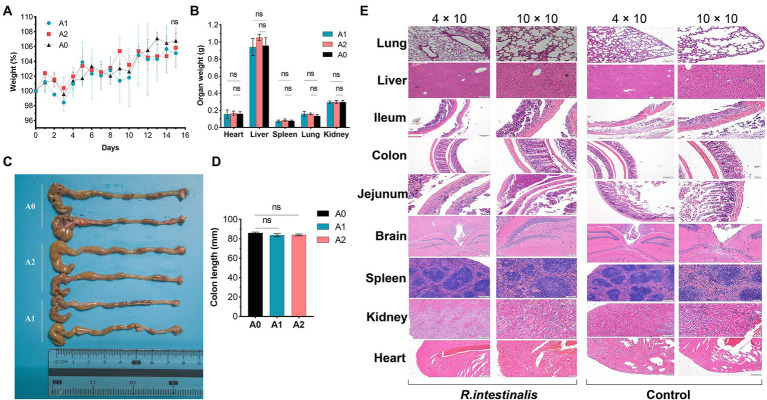
Acute toxicity study. **(A)** Body weight. **(B)** Weight of vital organs (heart, lung, liver, spleen, kidney). **(C,D)** Colon length. **(E)** Hematoxylin–eosin staining pathological examination results of vital organs. * indicates *p* < 0.05, ns indicates no significance.

During observation period, no mortality, morbidity, or abnormal clinical manifestations were observed. The average daily food and water intake is presented in Supplementary Tables S3, S4. Body weight, organ weight, and colon length were not significantly different between the experimental and control groups (Figures 9A–D). The organs exhibited no obvious pathological damage such as necrosis, inflammation, or proliferation (Figure 9E). All hematological parameters in the mice were comparable between the groups (Supplementary Table S5). There were no significant differences between the experimental and control groups in terms of alanine aminotransferase, aspartate aminotransferase, γ-glutamyltransferase, total bile acid, and creatinine (Table 4). Notably, serum uric acid levels in the experimental group were significantly lower than those in the control group, suggesting that *R. intestinalis* may affect purine and uric acid metabolism. Based on these results, we determined that oral *R. intestinalis* has an LD50 exceeding 1.9 × 10^9^ CFU/kg.

**Table 4:** Clinical chemistry results of acute toxicity study.

	*R. intestinalis*	Control	*P*
ALT (U/L)	41.29 ± 8.1	36.88 ± 7.73	0.420
ALP (U/L)	136.73 ± 17.32	188.22 ± 15.79	0.105
AST (U/L)	121.44 ± 18.89	174.17 ± 82.18	0.667
γ-GT (U/L)	0.89 ± 0.85	1.92 ± 1.56	0.744
UREA (mg/dL)	32.35 ± 2.35	35.07 ± 2.41	0.317
CREA (umol/L)	13.14 ± 0.81	13.43 ± 0.19	0.330
DBIL (umol/L)	4.04 ± 0.41	9.19 ± 5.05	0.439
TBA (umol/L)	3.27 ± 0.34	4.7 ± 0.5	0.443
UA (umol/L)	75.58 ± 1.62	163.22 ± 26.18	0.035
TBIL (umol/L)	8.33 ± 0.68	15.77 ± 8.14	0.549

ALT: Alanine aminotransferase; AST: Aspartate aminotransferase; ALP: alkaline phosphatase; γ-GT: γ-glutamyltransferase; DBIL: Direct Bilirubin; TBA: total bile acid; TBIL: total bilirubin; UA: uric acid.

### 28-day repeated dose study

**Figure 10:**
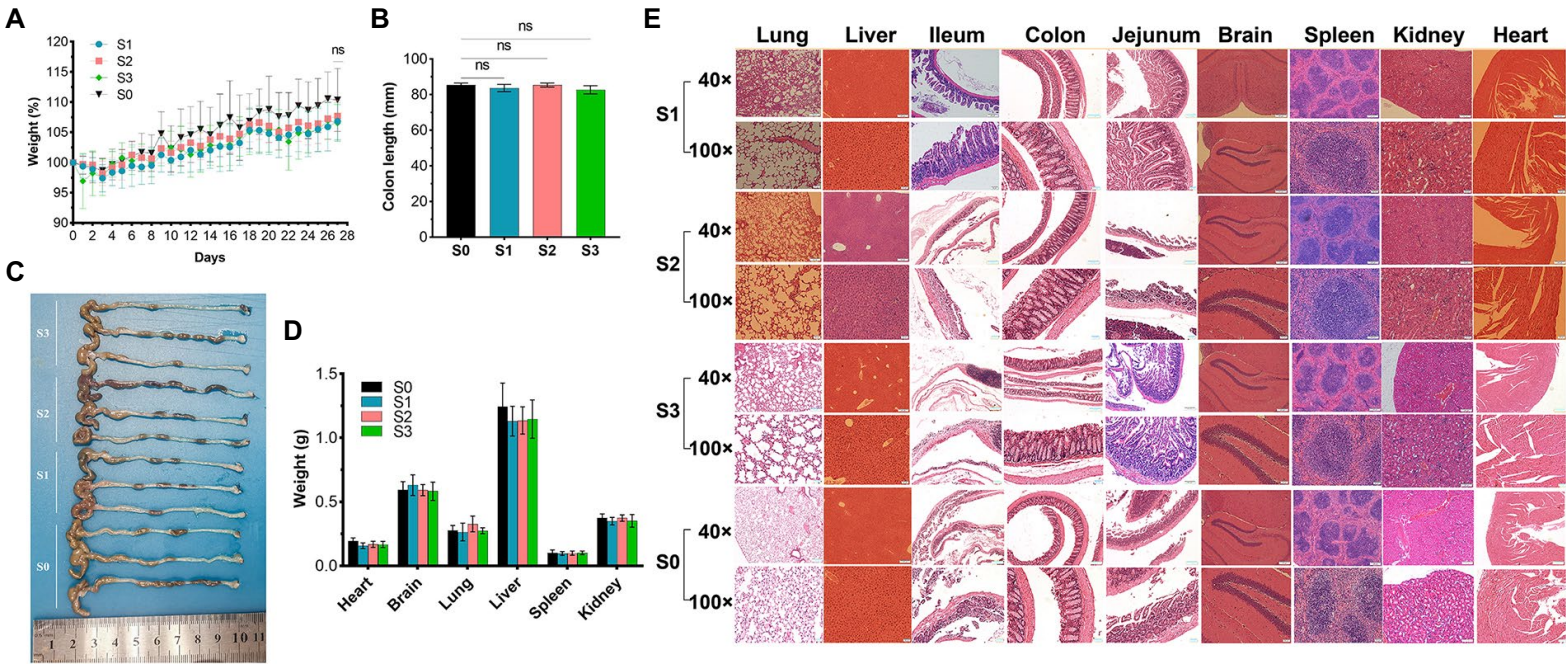
28-Day repeated dose study. **(A)** Body weight. **(B,C)** Colon length. **(D)** Weight of vital organs (heart, lung, brain, liver, spleen, kidney). **(E)** Hematoxylin–eosin staining pathological examination of organs. * indicates *p* < 0.05, ns indicates no significance.

No deaths or dosing-related adverse reactions were observed at any dose level during the 28-day experimental period. The average daily food and water intake is presented in Supplementary Tables S6, S7. Body weight, organ weight, and colon length did not differ significantly between groups (Figures 10A–D). No obvious pathological changes, such as necrosis, inflammation, or proliferation, were observed in the organs (Figure 10E). The hematological parameters did not differ significantly among the groups (Supplementary Table S8). In terms of alanine aminotransferase (ALT), aspartate aminotransferase (AST), alkaline phosphatase (ALP), γ-glutamyltransferase (γ-GT), total bile acid (TBA), and urea levels (Table 5), no significant differences were observed between the groups. Notably, the serum uric acid level in the experimental group was significantly lower than that in the control group, in agreement with the results of the acute oral toxicity test. Based on these data, the NOAEL for the oral administration of *R. intestinalis* was estimated at 1.32 × 10^9^ CFU/kg/day for 28 days.

**Table 5:** Clinical chemistry results of 28-day repeated dose study.

	S0	S1	S2	S3	*P*
AST (U/L)	176.06 ± 57.97	229.68 ± 103.16	149.19 ± 23.92	145.71 ± 46.66	0.28
ALT (U/L)	42.06 ± 12.43	33.92 ± 4.48	36.4 ± 8.89	49.5 ± 11.26	0.17
DBIL (umol/L)	3.99 ± 0.75	5.11 ± 1.72	3.66 ± 0.39	3.91 ± 1.19	0.32
TBIL (umol/L)	11.02 ± 1.55	8.73 ± 1.01	8.35 ± 0.98	9.35 ± 1.45	0.05
ALP (U/L)	154.2 ± 19.17	140.39 ± 25.04	164.5 ± 29.73	140.22 ± 26.72	0.49
γGT (U/L)	1.92 ± 1.37	2.26 ± 0.83	2.62 ± 2.86	2.12 ± 0.5	0.94
TBA (umol/L)	4.73 ± 1.46	22.88 ± 36.04	6.38 ± 4.6	4.73 ± 3.31	0.45
UREA (umol/L)	26.4 ± 1.97	23.6 ± 3.19	25.07 ± 1.36	25.81 ± 2.64	0.42
CREA (umol/L)	26.83 ± 0.51	25.85 ± 1.75	23.98 ± 2.56	29.14 ± 6.19	0.26
UA (umol/L)	115.93 ± 36.31	107.9 ± 17.82	69.49 ± 13.33	69.66 ± 22.83	0.03

## Discussion

Genomic, phenotypic, and oral safety studies should be conducted on potential probiotic strains to assess their safety and feasibility for industrial use.

A few virulence factors (VFs) have also been found in the genomes of nonpathogenic bacteria (Niu et al., 2013). Certain VFs are involved in host–microbe interactions, while others are involved in cell adhesion and host defense (Li et al., 2018). VF genes found in the *R. intestinalis* genome were related to adhesion (*fbpA/fbp68*, *ebpC*, *efaA*, *plr*/*gapA*) and lipid and fatty acid metabolism (*panD*). *FbpA* may play a role in the prevention of pathogenic colonization by inhibiting biofilm formation (Wang et al., 2017). *EfaA* is associated with adhesion of probiotics bacteria to both biotic and abiotic surfaces (Choeisoongnern et al., 2021). *GroEL* may inhibit colitis by inhibiting pro-inflammatory M1 macrophages and promoting the secretion of anti-inflammatory cytokines (Dias et al., 2021). The genome contains two tetracycline resistance genes, but a subsequent antibiotic susceptibility test showed that *R. intestinalis* was susceptible to tetracycline. Gene modifications or pseudogenes may have causes this seemingly paradoxical result. Additionally, the expression of these genes may usually be low or they are induced only under specific circumstances, such as stimuli or signals from the environment (Li et al., 2018). Therefore, the presence of these resistance genes does not hinder the safety of *R. intestinalis* as a probiotic.

The CRISPR-Cas system is considered an defense mechanism against mobile genetic elements (Holý et al., 2020). This system can protect against re-invasion by capturing and integrating foreign nucleic acid fragments from the initial invasion. CRISPR-Cas genes have also been found in some probiotics, such as *Bifidobacterium* (Pan et al., 2020) and *Lactobacillus reuteri* (Alayande et al., 2020). Amino acids can be converted to biogenic amines *via* microbial decarboxylation. Histamine and tyramine (Zhu et al., 2020) are prevalent in fermented foods, and the excessive intake of biogenic amines can be harmful. The *R. intestinalis* genome contained genes involved in spermidine biosynthesis. Spermidine improves gut barrier integrity (Ma et al., 2020b), reduces obesity and cancer mortality, and provides anti-inflammatory and stem cell senescence protection (Madeo et al., 2018). Probiotics, including *Akkermansia muciniphila*, may have anti-aging and anti-obesity effects, that are linked to increased levels of spermidine in the host (Grajeda-Iglesias et al., 2021).

Microbes in the gut synthesize several vitamins and essential amino acids that contribute to the host amino acid homeostasis (Gill et al., 2006; Lin et al., 2017). *R. intestinalis* contains genes for the biosynthesis of five essential amino acids and five vitamins. Prophages are generally considered beneficial to their bacterial hosts, especially in the gastrointestinal environment (Manrique et al., 2017). The presence of prophages not only increases genetic variability but may also allow bacteria to cope with adverse environmental conditions (Casjens, 2003). Several probiotics contain prophages, including *Lactococcus*, *Bifidobacterium*, *Lactobacillus* (Pei et al., 2021), and *L. rhamnosus* (Brandt et al., 2001). Additionally, studies have suggested that prophages are present in more than 92% of *Lactobacillus* genomes (Sun et al., 2015). Jeffrey et al. (Cornuault et al., 2020) identified two active prophages in the genome of *R. intestinalis* and demonstrated that these prophages can influence short-term changes in the gut microbiota composition.

Bacteriocins are products synthesized by bacterial ribosomes that possess bacteriostatic properties (Deraz et al., 2005). Increasing antibiotic resistance has prompted researchers to focus on bacteriocins, since these may serve as alternatives to antibiotics (Cotter et al., 2013). *R. intestinalis* can produce Zoocin A, a 30 kDa D-alanyl-L-alanine endopeptidase bacteriocin that inhibits streptococcal growth by binding and cleaving bacterial peptidoglycan (Gargis et al., 2009). *L. plantarum* (Seddik et al., 2017) and *Enterococcus* spp. (Ben Braïek and Smaoui, 2019) are two probiotics microbes known to produce bacteriocins. *R. intestinalis* contains four short-chain fatty acid (SCFA) synthesis gene clusters, including a butyrate synthesis gene cluster. Butyrate has several beneficial effects, including as an energy source for colonic epithelial cells and exerting anti-inflammatory properties by inhibiting nuclear factor (NF)-κB (Segain et al., 2000). *Clostridium butyricum*, a butyrate-producing probiotic, inhibits intestinal tumor growth by modulating the Wnt pathway and gut microbiota (Chen et al., 2020). Moreover, VSL#3 modulates the gut microbiota-SCFA-hormone axis to combat obesity and diabetes (Yadav et al., 2013).

As the human genome encodes only approximately 17 carbohydrate-degrading enzymes, those produced by gut bacteria play a critical role in carbohydrate degradation (Bhattacharya et al., 2015). *R. intestinalis* contains 222 carbohydrate metabolism-related genes that likely play important roles in host carbohydrate metabolism. Maria et al. (Leth et al., 2020) demonstrated that *R. intestinalis* expresses RiCBM86, an enzyme that binds and absorbs xylan in the gut, reduces inflammation, and improves atherosclerosis (Kasahara et al., 2018). This bacterium is also a major degrader of β-mannans (La Rosa et al., 2019) and can produce xylanase to degrade xylan (Mirande et al., 2010). GIs are an important aspect of horizontal gene transfer and can transmit antibiotic resistance and virulence genes (Juhas et al., 2009). *R. intestinalis* contains 41 GIs, none of which contain virulence, antibiotic resistance, or pathogenicity genes.

Although the gastric juice pH is 1–3, it increases to 6 after food ingestion (De Angelis et al., 2006). The pH values of the duodenum, terminal ileum, cecum, and rectum are 6.0, 7.4, 5.7, and 6.7, respectively (Fallingborg, 1999). Several methods have been reported for enhancing the tolerance of probiotics to bile salts and acids. Gou et al. (2021) used soybean lecithin and whey protein concentrate to treat *Lacticaseibacillus paracasei* and successfully increased its tolerance to bile salts and acids. Evidence suggests that 5% lactose can enhance the bile salt tolerance of *L. bulgaricus* (Mena and Aryana, 2018), whereas soy lecithin (Hu et al., 2015) can enhance the bile salt resistance of *L. plantarum* and whey protein (Vargas et al., 2015) can enhance the acid and bile salt tolerance of *Streptococcus thermophilus*. Biofilm formation by probiotics can inhibit pathogens from colonizing the mucosa (Deng et al., 2020). *R. intestinalis* forms a higher percentage of biofilms than many common probiotics and pathogenic bacteria, further highlighting its probiotic properties.

Following the administration of *R. intestinalis*, the serum uric acid level decreased significantly, suggesting that the bacterium may influence the host’s purine and uric acid metabolism. Our *in vitro* study indicates that *R. intestinalis* can degrade 60% of the uric acid in the surrounding environment within 24 h (data not shown), although further investigation is required to determine the underlying mechanism.

*R. intestinalis* did not adversely affect the abundance or variety of the gut flora. After 14 days of continuous administration, the relative abundance of *R. intestinalis* did not increase proportionally. However, the structure of the flora changed, and the predominant flora consisted of *Barnesiella* and *porphyromonadaceae*. The relative abundance of *R. intestinalis* was determined by 16S rRNA sequencing of stool samples. Owing to their ease of collection, feces are often used to study the gut microbiota. Nonetheless, this method has a limited ability to detect the mucosal-associated microbiota. Mucosal-associated microbiota and luminal microbiota have only a partial association (Zmora et al., 2018). Due to their biofilm-like structure, mucosal-associated microbiota promote beneficial functions in intestinal epithelial cells (Sonnenburg et al., 2004), and conventional sampling methods (feces or intestinal tissue) may underestimate their levels (Donaldson et al., 2016). *R. intestinalis* adheres to the intestinal mucosa and colonizes the cecum and colonic mucosa. Flow cytometry results showed that there was a low abundance of *R. intestinalis* in the intestinal contents (Figure 8B). Similarly, some probiotics, such as *B. lactis*, showed a decreasing trend in its levels after prolonged administration (Ma et al., 2020a). Therefore, we speculated that the variation in the gut flora is stratified rather than continuous, and a relatively balanced state of host-microbe symbiosis exists in the gut microbiota (Arumugam et al., 2011). Studies indicate that the gut microbiota is partially stable, with approximately 40 species of bacteria forming a core microbiota that persists in humans for at least 1 year (Donaldson et al., 2016). Additionally, mucosal flora analysis revealed an increase in *Enterobacteriaceae* and a decrease in *Clostridium* in patients with Crohn’s disease. However, these features disappeared when stool samples were examined (Gevers et al., 2014).

Probiotics may have an impact on the host even though they do not necessarily interact with indigenous microbiota (Hill et al., 2014). Instead, they may normalize the disturbed microbiota and modulate it in a beneficial manner. It is difficult to identify patterns of change in commonly altered microbes among studies that report probiotic-associated microbiome alterations. The average relative abundance of *R. intestinalis* in healthy individuals is estimated at only 0.09377% according to the Gmrepo database (Dai et al., 2022). Thus, *R. intestinalis* is not predominant in the entire intestinal flora composition. This may explain why, after taking *R. intestinalis* for a short period, it remains difficult for the bacterium to become the predominant. A clinical study also found that after administration *L. paracasei* DG, the α-diversity of the intestinal flora did not change, but the β-diversity and structure changed (Ferrario et al., 2014) with an increase in *Brucella* and *Faecalis*. Administration of *R. intestinalis* increased the levels of certain beneficial gut bacteria. *Porphyromonadaceae* abundance increased in long-lived populations, and the proportion of *R. intestinalis* also increased (Ren et al., 2021). The abundance of *Barnesiella*, *Porphyromonadaceae*, and *Roseburia* also increased after vitamin D1 administration in patients with Crohn’s disease (Schäffler et al., 2018), indicating that these bacteria may function synergistically. In addition to its resistance to IBD (Weiss et al., 2014), *Barnesiella* is a valuable “oncomicrobiotic” for antitumor immunomodulator therapy (Daillère et al., 2016). *Barnesiella* has also been associated with remission from obesity and hepatic steatosis (Rodriguez et al., 2020) and with the clearance of vancomycin-resistant *Enterococcus* in the gut (Ubeda et al., 2013). *Porphyromonadaceae* has been associated with reduced visceral fat and a healthier metabolic profile in the elderly (Tavella et al., 2021). Therefore, the probiotic effect of *R. intestinalis* may be that it constitutes an interaction network with several beneficial gut microbes, such as *Barnesiella* and *Porphyromonadaceae*.

CFDA-SE is a fluorescent dye with many advantages such as strong fluorescence, low toxicity, and good stability. Several researchers (Lee et al., 2004; Wang et al., 2019, 2021; Zhao et al., 2022) have used this method to tag and track bacteria. *R. intestinalis* was detected in all intestinal sites 2 h after administration, whereas after 48 h, *R. intestinalis* was predominantly located in the cecum, and not present in the colon. This distribution may be related to the physiology of the different parts of the gut (Donaldson et al., 2016; Martinez-Guryn et al., 2019). Using frozen section analysis, we observed that *R. intestinalis* colonizing the cecum and mucus layer of the colon. Abbeele et al. demonstrated that *R. intestinalis* colonized the mucin layer using *in vitro* intestinal models (Van den Abbeele et al., 2013), which corresponds with our observations *in vivo*.

## Conclusion

In conclusion, we performed a comprehensive safety assessment of *R. intestinalis*. An *in vitro* study and genomic analysis revealed that *R. intestinalis* was not cytotoxic and caused no safety concerns associated with antibiotic resistance genes, virulence factors, biogenic amine production, gelatinase, or DNase activity. *In vivo* experiments showed that orally administrated *R. intestinalis* mainly colonizes the cecum and colonic mucus layers without altering the abundance and diversity of the gut microbiota. An oral toxicity study conducted in mice revealed that *R. intestinalis* was not toxic and could reduce serum uric acid levels. This study highlights *R. intestinalis* as a non-pathogenic strain suitable for use as a “Next Generation Probiotic.”

## Data availability statement

The raw sequences generated for this study can be found in the NCBI Short Read Archive under BioProject no. PRJNA850191.

## Ethics statement

The animal study was reviewed and approved by Central South University Animal Ethics Committee (XMSB-2022-0198).

## Author contributions

CZ analyzed the strain phenotypically and genetically and drafted the manuscript. KM and KN helped with genomic and bioinformatic analyses. WL and XinW contributed to the phenotypic experiments. YH helped to carry out the experiments on mice and fluorescently label of bacteria. MD contributed to revising the manuscript. XiaW oversaw the project and reviewed and revised the manuscript. All authors contributed to the article and approved the submitted version.

## Funding

This project was supported by the National Natural Science Foundation of China (NSFC no. 81970494).

## Conflict of interest

The authors declare that the research was conducted in the absence of any commercial or financial relationships that could be construed as a potential conflict of interest.

## Publisher’s note

All claims expressed in this article are solely those of the authors and do not necessarily represent those of their affiliated organizations, or those of the publisher, the editors and the reviewers. Any product that may be evaluated in this article, or claim that may be made by its manufacturer, is not guaranteed or endorsed by the publisher.

## Supplementary material

The Supplementary material for this article can be found online at: https://www.frontiersin.org/articles/10.3389/fmicb.2022.973046/full#supplementary-material

## Footnotes

^1^ https://www.marketsandmarkets.com/PressReleases/probiotics.asp
