# Supplementary material for: Assessment of the safety and probiotic properties of Roseburia intestinalis: A potential “Next Generation Probiotic”
Source: Front Microbiol. 2022 Sep 8;13:973046. doi: 10.3389/fmicb.2022.973046 (PMC9493362; doi:10.3389/fmicb.2022.973046)
Supplement: Supplementary file 1 [file Table_1.DOCX]

Supplementary Material

# Supplementary Figures and Tables

##
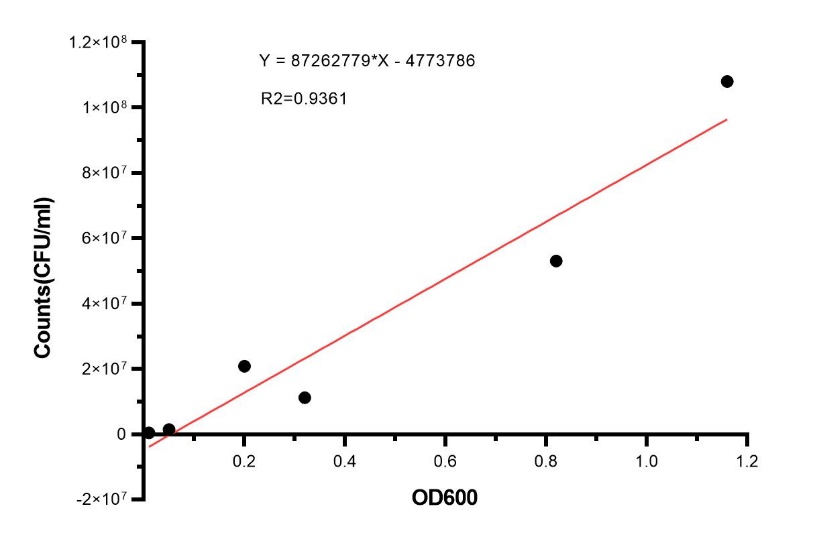
Supplementary Figures

**
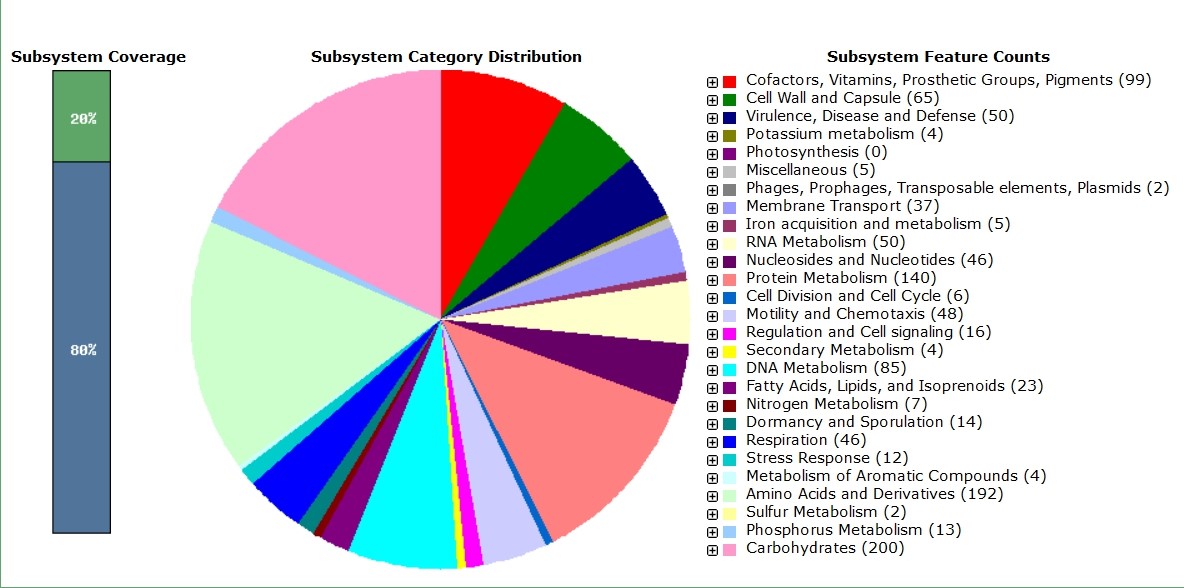
Supplementary Figure S1.** The standard curve of OD600 and corresponding CFU counts for *R.intestinalis* grown in BHI medium.

**Supplementary Figure S2.** Subsystem distribution of *R. intestinalis* on RAST annotation server. Out of 4340 coding sequences predicted by RAST, the subsystem coverage is 20% which contributes to a total of 252 subsystems.


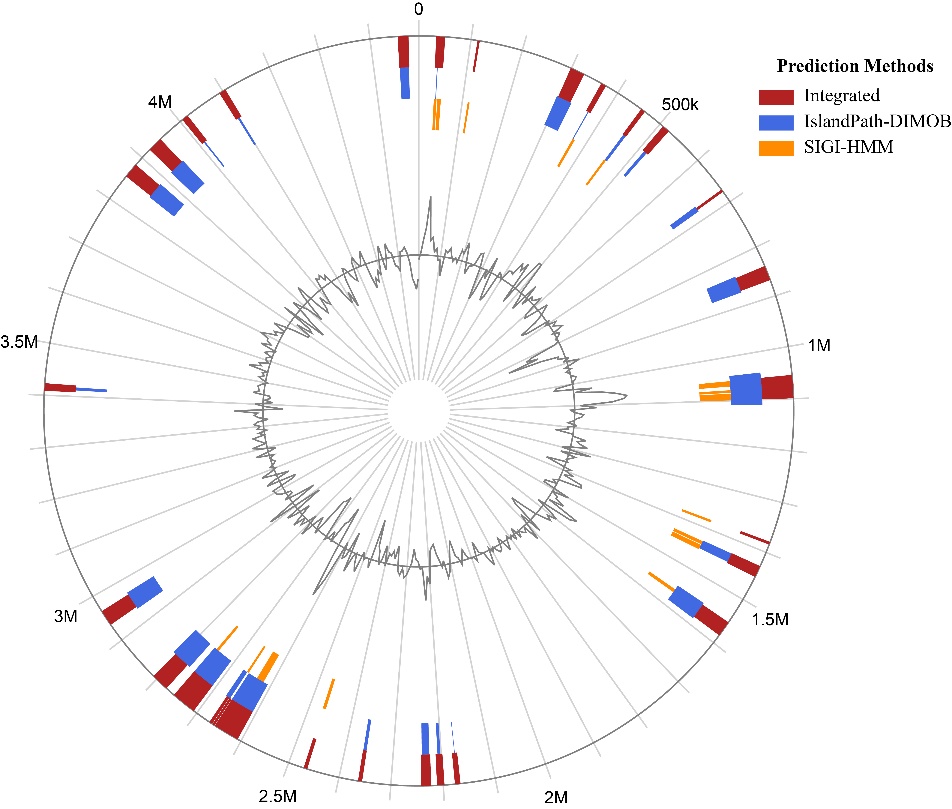


**
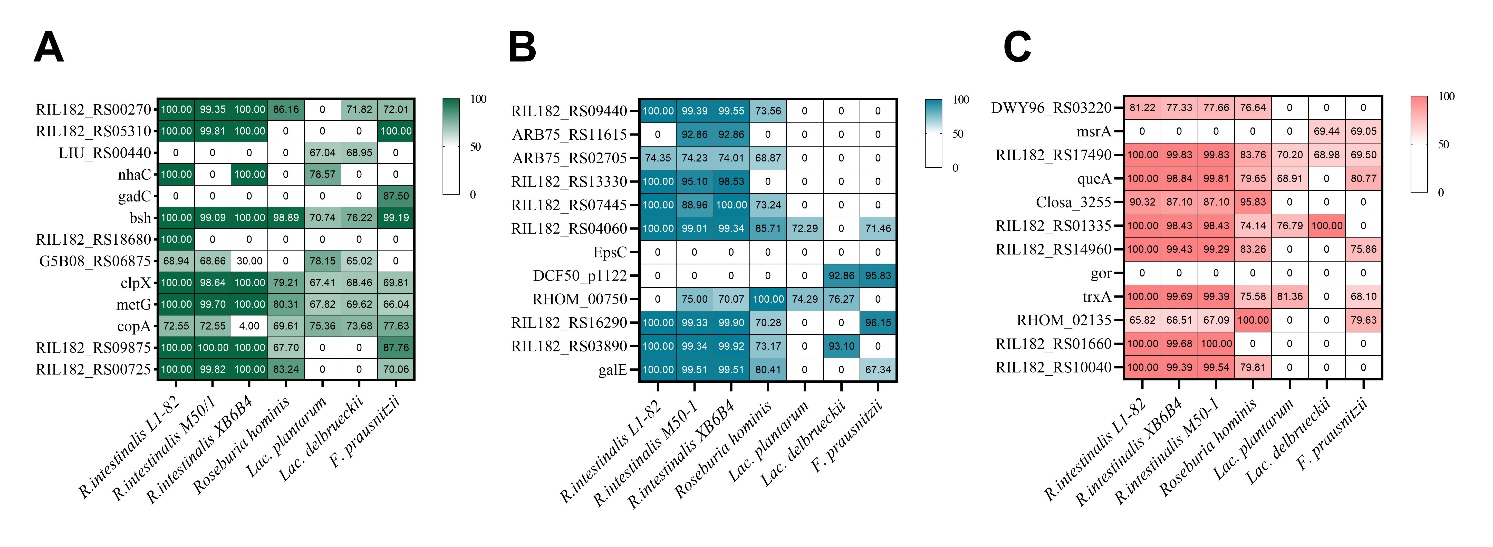
Supplementary Figure S3.** Genomic islands (GIs) within genome of *R. intestinalis*. Different colors represent GIs predicted using different methods. None of the annotated virulence genes, antibiotic resistance genes, or pathogenicity-related genes were found in the predicted GIs.

**Supplementary Figure S4.** Heatmap showing the clustering of the compared *R. intestinalis* L1-82 genomes based on the presence of **(A)** genes associated with survival in the GIT, **(B)** genes associated with adherence to the GIT, and **(C)** genes associated with antioxidant activity. The numbers in the figure represent the percent identity in the BLAST alignment. GraphPad Prism was used to create a heatmap of the analysis results in order to visualize the presence or absence of the genes.


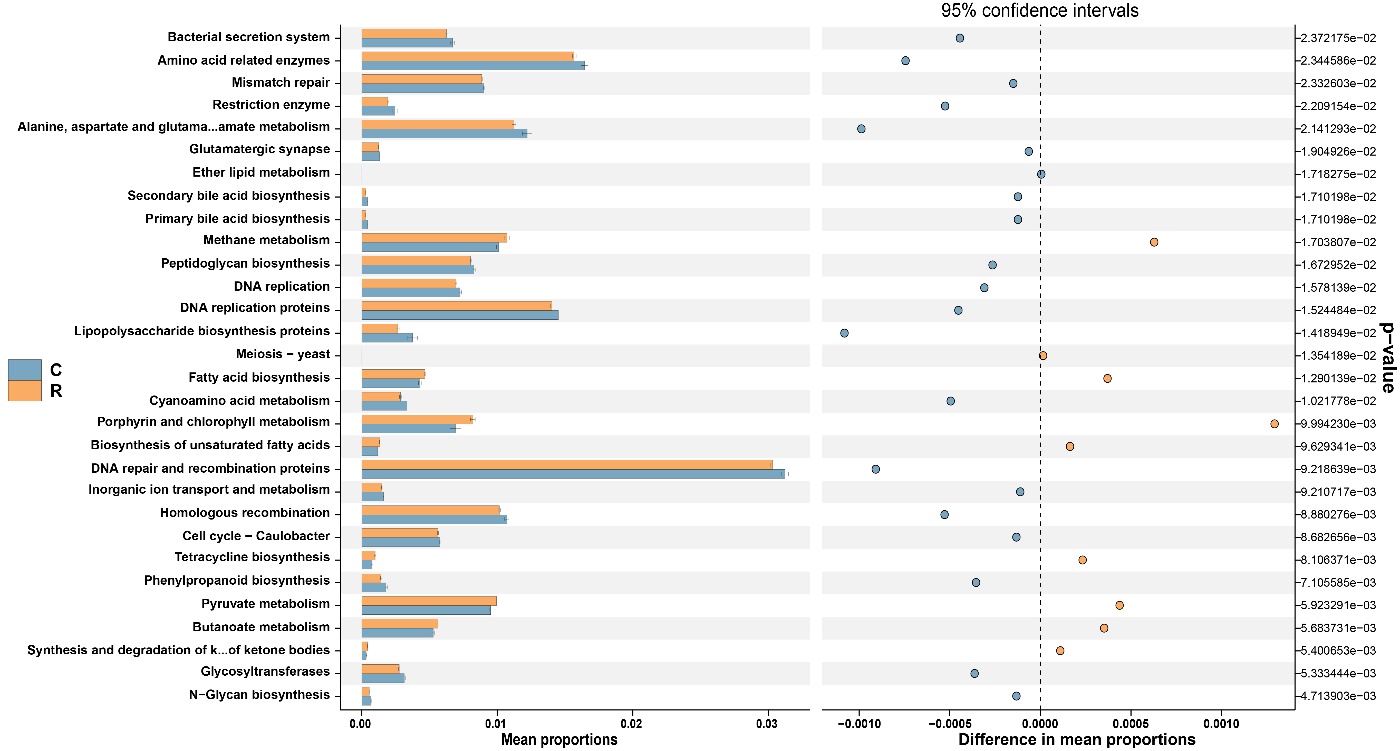

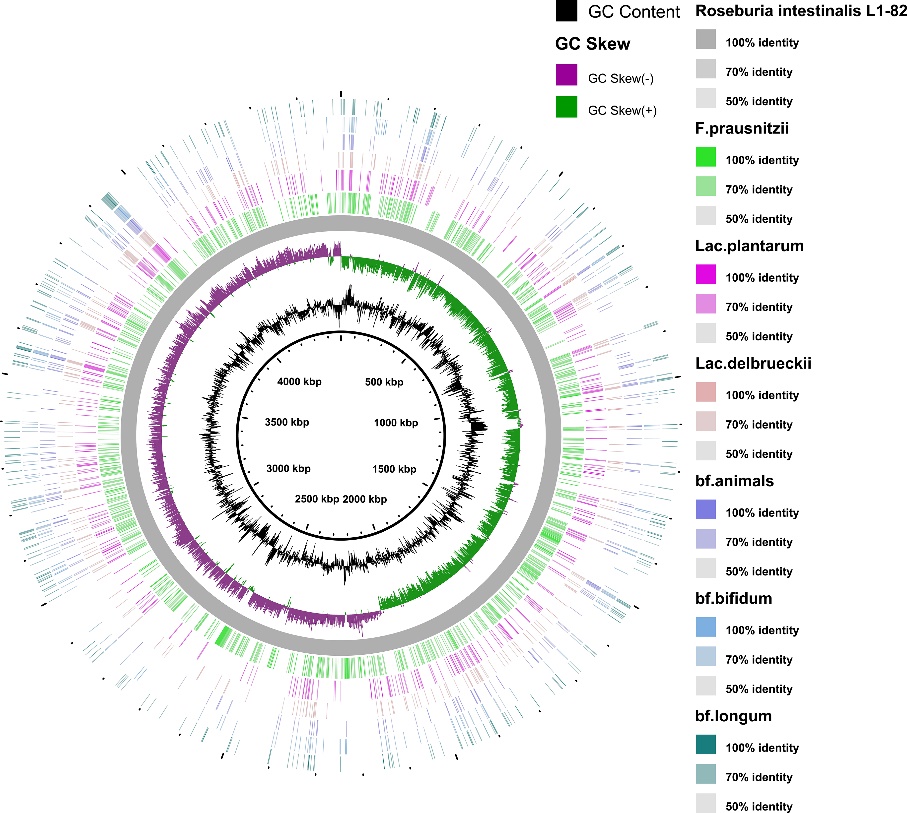
**Supplementary Figure S5.** Blast Ring Image Generator (BRIG) diagram comparing *R. intestinalis* genome with genomes of six potentially probiotics of IBD using genome of *R. intestinalis* as a reference.

**Supplementary Figure S6.** The PICRUSt2 tool based on the KEGG database was used to predict pathway enrichment and we observed differences in pathway enrichment after administration of *R. intestinalis*. C, Control group; R, *R. roseburia* group.


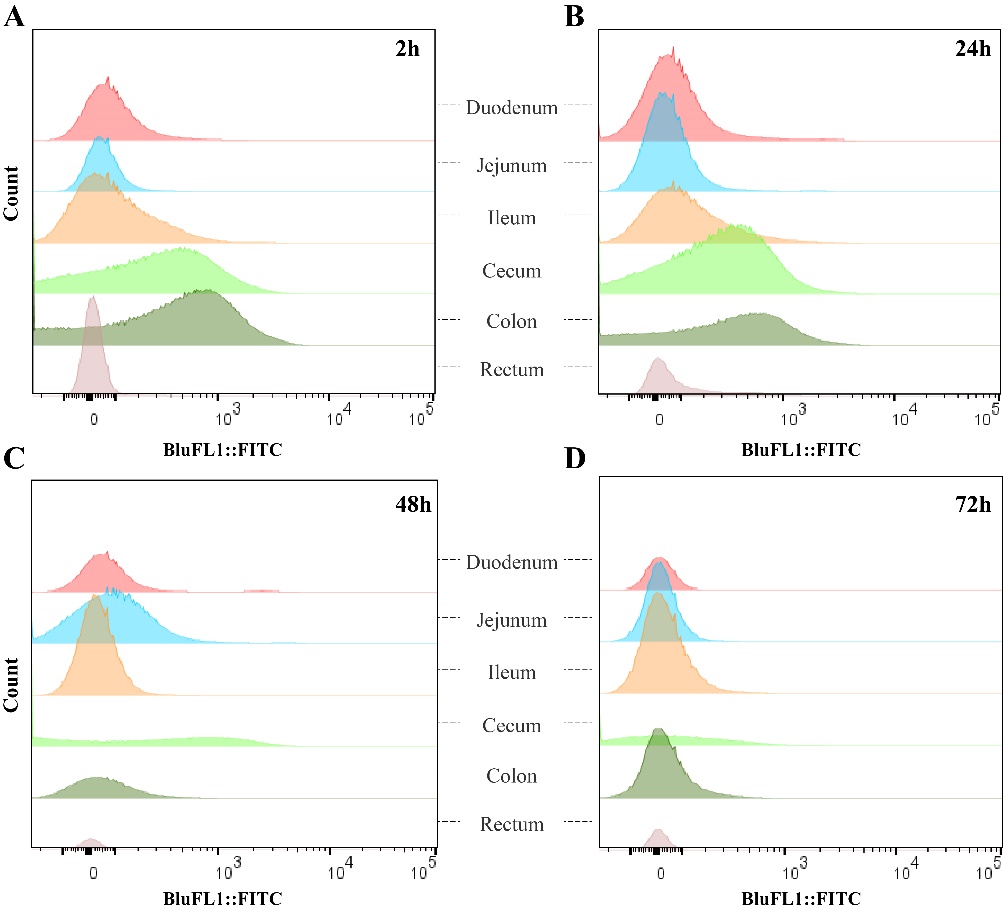


**Supplementary Figure S7.** Representative flow cytometry images of *R. intestinalis* labeled with CFDA-SE.

## Supplementary Tables

**Tsble S1.** The genomes of the *Roseburia* genus and several potential probiotic or pathogenic bacteria for inflammatory bowel disease

| **NCBI strain**  **identifiers** | **Lineage**  **(phylum; class)** | **GenBank**  **accession** | **Genome**  **Size (Mb)** | **GC%** | **CDS** |
| --- | --- | --- | --- | --- | --- |
| ***Roseburia* genus** |  |  |  |  |  |
| *R. intestinalis L1-82* | Firmicutes; Clostridia | GCA_900537995.1 | 4.49 | 42.7% | 4165 |
| *R. intestinalis M50/1* | Firmicutes; Clostridia | GCA_000209995 | 4.10 | 42% | 3656 |
| *R. intestinalis XB6B4* | Firmicutes; Clostridia | GCA_000210655.1 | 4.30 | 42.5% | 3737 |
| *Roseburia faecis* | Firmicutes; Clostridia | GCA_001405615.1 | 3.60 | 42.5% | 3418 |
| *Roseburia hominis* | Firmicutes; Clostridia | GCA_902387955.1 | 3.60 | 48% | 3191 |
| **Potential probiotic** |  |  |  |  |  |
| *Bifidobacterium longum* | Actinobacteria; Actinomycetia | GCA_000196555.1 | 2.39 | 60.3% | 2023 |
| *Bifidobacterium bifidum* | Actinobacteria; Actinomycetia | GCA_000273525.1 | 2.22 | 62.8% | 1864 |
| *Bifidobacterium animalis* | Actinobacteria; Actinomycetia | GCA_000224965.2 | 1.94 | 60.5% | 1613 |
| *Lactiplantibacillus plantarum* | Firmicutes; Bacilli | GCA_003269405.1 | 3.23 | 44.6% | 3011 |
| *Lactobacillus delbrueckii* | Firmicutes; Bacilli | GCA_001888925.1 | 1.89 | 50.1% | 1944 |
| *Faecalibacterium prausnitzii* | Firmicutes; Clostridia | GCA_003312465.1 | 2.97 | 56.4% | 2812 |
| *Escherichia coli Nissle 1917* | Proteobacteria; Gammaproteobacteria | GCA_021559835.1 | 5.10 | 50.5% | 4531 |
| **Potentially pathogenic** |  |  |  |  |  |
| *Escherichia coli LF82* | Proteobacteria; Gammaproteobacteria | GCA_000284495.1 | 4.80 | 50.5% | 4397 |
| *Enterococcus faecalis* | Firmicutes; Bacilli | GCA_000393015.1 | 2.87 | 37.5% | 2738 |
| *clostridium difficile* | Firmicutes; Clostridia | GCA_018885085.1 | 4.09 | 28.5% | 3715 |

Abbreviation: NCBI, National Center for Biotechnology Information

**Table S2.** Prophages found in the genome of *R.intestinalis* predicted by PHASTER

| **Region** | **Length** | **Completeness** | **Total proteins** | **Start** | **End** | **GC%** |
| --- | --- | --- | --- | --- | --- | --- |
| 1 | 7.6kb | questionable | 10 | 323386 | 331059 | 43.54% |
| 2 | 25.1 kb | incomplete | 16 | 455706 | 480832 | 44.10% |
| 3 | 18.1 kb | questionable | 17 | 849972 | 868121 | 40.25% |
| 4 | 47 kb | questionable | 62 | 1055116 | 1102120 | 51.38% |
| 5 | 21 kb | questionable | 29 | 2422078 | 2443160 | 42.26% |
| 6 | 10.8 kb | incomplete | 8 | 2450006 | 2460811 | 39.53% |
| 7 | 20.2 kb | incomplete | 11 | 2705147 | 2725440 | 36.85% |

**Table S3.** The average daily food consumption per mice in acute oral toxicity study

| Days | 0 | 1 | 2 | 3 | 4 | 5 | 6 | 7 | 8 | 9 | 10 | 11 | 12 | 13 | 14 |
| --- | --- | --- | --- | --- | --- | --- | --- | --- | --- | --- | --- | --- | --- | --- | --- |
| A1 | 3.43g | 3.10g | 3.47g | 3.87g | 3.67g | 3.07g | 3.27g | 3.63g | 3.43g | 3.60g | 4.27g | 3.13g | 3.57g | 3.47g | 3.50g |
| A2 | 3.97g | 4.47g | 4.43g | 4.37g | 3.87g | 3.73g | 3.83g | 4.07g | 4.73g | 4.07g | 4.37g | 3.60g | 3.93g | 4.03g | 4.60g |
| A0 | 3.28g | 3.23g | 3.37g | 3.65g | 3.05g | 3.45g | 3.37g | 3.23g | 3.83g | 3.17g | 3.50g | 3.47g | 3.43g | 3.18g | 2.85g |

**Table S4.** Average daily water consumption per mice in acute oral toxicity study

| Days | 0 | 1 | 2 | 3 | 4 | 5 | 6 | 7 | 8 | 9 | 10 | 11 | 12 | 13 | 14 |
| --- | --- | --- | --- | --- | --- | --- | --- | --- | --- | --- | --- | --- | --- | --- | --- |
| A1 | 6.00ml | 5.33ml | 4.33ml | 4.67ml | 6.67ml | 4.33ml | 5.33ml | 4.67ml | 6.00ml | 5.00ml | 5.67ml | 6.33ml | 5.33ml | 4.00ml | 6.00ml |
| A2 | 6.00ml | 6.67ml | 7.33ml | 6.00ml | 7.50ml | 7.33ml | 6.00ml | 7.50ml | 6.67ml | 5.33ml | 6.67ml | 5.00ml | 6.00ml | 5.00ml | 8.33ml |
| A0 | 5.33ml | 5.83ml | 5.67ml | 4.67ml | 4.83ml | 3.83ml | 4.50ml | 4.67ml | 5.00ml | 5.67ml | 4.67ml | 4.67ml | 4.83ml | 4.33ml | 4.50ml |

**Table S5.** Hematology results of acute toxicity study

|  |  | ***R. intestinalis*** | **Control** | ***P*** |
| --- | --- | --- | --- | --- |
| WBC | 10^9^/L | 2.43 ± 0.99 | 2.3 ± 0.22 | 0.861 |
| Lymph# | 10^9^/L | 1.5 ± 0.29 | 1.67 ± 0.25 | 0.574 |
| Mon# | 10^9^/L | 0.1 ± 0.08 | 0.1 ± 0.03 | 1.000 |
| Gran# | 10^9^/L | 0.83 ± 0.62 | 0.53 ± 0.05 | 0.564 |
| Lymph% | % | 65.53 ± 10.37 | 72.37 ± 4.15 | 0.436 |
| Mon% | % | 3.8 ± 0.88 | 3.2 ± 0.24 | 0.407 |
| Gran% | % | 30.67 ± 9.99 | 24.43 ± 4.11 | 0.460 |
| RBC | 10^12^/L | 9.52 ± 0.06 | 9.55 ± 0.23 | 0.854 |
| HGB | g/L | 143 ± 2.94 | 144 ± 4.08 | 0.793 |
| HCT | % | 44.93 ± 1.43 | 45.47 ± 1.15 | 0.702 |
| MCV | fL | 47.27 ± 1.18 | 47.67 ± 0.09 | 0.659 |
| MCH | pg | 14.97 ± 0.24 | 15.03 ± 0.05 | 0.730 |
| MCHC | g/L | 317.67 ± 4.5 | 316 ± 1.41 | 0.643 |
| RDW | % | 16.2 ± 0.14 | 16.43 ± 0.19 | 0.234 |
| PLT | 10^9^/L | 1479.67 ± 253.85 | 1370 ± 86.48 | 0.594 |
| MPV | fL | 5.8 ± 0.28 | 5.8 ± 0.14 | 1.000 |
| PDW |  | 16.17 ± 0.05 | 16.1 ± 0.08 | 0.374 |

**Table S6.** The average daily food consumption per mice in 28-Day repeated dose study

| **Days** | **S1** | **S2** | **S3** | **S0** | **Days** | **S1** | **S2** | **S3** | **S0** |
| --- | --- | --- | --- | --- | --- | --- | --- | --- | --- |
| **0** | 4.08 g | 4.27 g | 2.78 g | 4.25 g | **14** | 2.95 g | 3.88 g | 2.98 g | 4.50 g |
| **1** | 3.85 g | 4.17 g | 3.57 g | 4.42 g | **15** | 2.83 g | 3.15 g | 2.93 g | 4.17 g |
| **2** | 4.15 g | 4.17 g | 3.80 g | 4.35 g | **16** | 3.10 g | 3.87 g | 3.05 g | 4.25 g |
| **3** | 3.78 g | 4.55 g | 3.72 g | 4.77 g | **17** | 3.42 g | 3.62 g | 3.20 g | 3.88 g |
| **4** | 3.23 g | 3.57 g | 4.05 g | 4.25 g | **18** | 3.27 g | 3.42 g | 3.30 g | 3.93 g |
| **5** | 3.45 g | 4.10 g | 3.68 g | 4.45 g | **19** | 3.52 g | 3.47 g | 3.38 g | 4.43 g |
| **6** | 3.35 g | 4.35 g | 3.88 g | 4.82 g | **20** | 3.60 g | 3.35 g | 3.50 g | 4.18 g |
| **7** | 3.35 g | 4.10 g | 3.62 g | 4.12 g | **21** | 3.50 g | 3.57 g | 2.88 g | 3.85 g |
| **8** | 4.08 g | 4.98 g | 4.43 g | 5.28 g | **22** | 3.22 g | 3.70 g | 3.13 g | 3.92 g |
| **9** | 3.47 g | 3.87 g | 3.22 g | 3.75 g | **23** | 3.37 g | 3.62 g | 3.47 g | 3.85 g |
| **10** | 3.68 g | 3.90 g | 3.37 g | 4.77 g | **24** | 3.42 g | 3.42 g | 3.52 g | 4.18 g |
| **11** | 3.27 g | 3.43 g | 2.88 g | 4.60 g | **25** | 3.27 g | 3.57 g | 3.77 g | 3.93 g |
| **12** | 3.57 g | 3.12 g | 3.12 g | 4.72 g | **26** | 3.43 g | 3.55 g | 3.23 g | 4.00 g |
| **13** | 3.12 g | 3.48 g | 2.87 g | 4.12 g |  |  |  |  |  |

**Table S7.** The average daily water intake per mice in 28-Day repeated dose study

| **Days** | **S1** | **S2** | **S3** | **S0** | **Days** | **S1** | **S2** | **S3** | **S0** |
| --- | --- | --- | --- | --- | --- | --- | --- | --- | --- |
| **0** | 5.42 ml | 4.83 ml | 3.75 ml | 6.67 ml | **14** | 3.50 ml | 4.17 ml | 4.50 ml | 5.83 ml |
| **1** | 3.75 ml | 3.33 ml | 4.17 ml | 5.83 ml | **15** | 3.83 ml | 4.00 ml | 4.17 ml | 7.00 ml |
| **2** | 3.75 ml | 5.00 ml | 5.67 ml | 8.17 ml | **16** | 4.67 ml | 5.33 ml | 6.00 ml | 7.50 ml |
| **3** | 4.17 ml | 3.67 ml | 3.50 ml | 4.17 ml | **17** | 4.17 ml | 4.33 ml | 4.00 ml | 6.00 ml |
| **4** | 4.00 ml | 4.67 ml | 5.42 ml | 5.00 ml | **18** | 5.00 ml | 5.00 ml | 5.50 ml | 6.67 ml |
| **5** | 4.33 ml | 4.83 ml | 4.67 ml | 4.33 ml | **19** | 5.33 ml | 5.67 ml | 4.33 ml | 6.17 ml |
| **6** | 4.67 ml | 5.00 ml | 4.83 ml | 4.33 ml | **20** | 5.00 ml | 4.17 ml | 4.50 ml | 6.33 ml |
| **7** | 4.17 ml | 5.00 ml | 4.67 ml | 6.33 ml | **21** | 4.17 ml | 5.33 ml | 3.33 ml | 6.17 ml |
| **8** | 3.83 ml | 4.33 ml | 4.67 ml | 7.83 ml | **22** | 5.00 ml | 4.67 ml | 4.50 ml | 6.50 ml |
| **9** | 6.00 ml | 5.33 ml | 4.83 ml | 5.33 ml | **23** | 5.17 ml | 5.00 ml | 4.67 ml | 7.83 ml |
| **10** | 4.00 ml | 4.67 ml | 4.83 ml | 6.00 ml | **24** | 5.17 ml | 5.17 ml | 4.33 ml | 6.17 ml |
| **11** | 4.17 ml | 4.50 ml | 4.17 ml | 5.83 ml | **25** | 4.83 ml | 5.83 ml | 5.00 ml | 5.50 ml |
| **12** | 5.17 ml | 4.67 ml | 4.17 ml | 6.17 ml | **26** | 4.33 ml | 4.17 ml | 4.17 ml | 6.33 ml |
| **13** | 4.67 ml | 3.83 ml | 3.50 ml | 5.67 ml |  |  |  |  |  |

**Table S8.** Hematology results of 28-Day repeated dose study

|  |  | **S0** | **S1** | **S2** | **S3** | ***P*** |
| --- | --- | --- | --- | --- | --- | --- |
| WBC | 10^9^/L | 2.1 ± 1.73 | 2.63 ± 1.19 | 2.8 ± 1.05 | 1.47 ± 0.55 | 0.56 |
| Lymph | 10^9^/L | 1.8 ± 1.48 | 2 ± 0.79 | 2.23 ± 0.87 | 1.13 ± 0.51 | 0.58 |
| Mon | 10^9^/L | 0.03 ± 0.06 | 0.07 ± 0.06 | 0.03 ± 0.06 | 0 ± 0 | 0.49 |
| Gran | 10^9^/L | 0.27 ± 0.21 | 0.57 ± 0.38 | 0.53 ± 0.21 | 0.33 ± 0.15 | 0.43 |
| Lymph1_A | % | 81.77 ± 9.16 | 76.43 ± 3.91 | 80.37 ± 4.16 | 70.8 ± 10.67 | 0.35 |
| Mon1_A | % | 2.57 ± 1.31 | 3.47 ± 1.27 | 2.4 ± 0.92 | 2.67 ± 0.78 | 0.65 |
| Gran1_A | % | 15.67 ± 7.96 | 20.1 ± 3.8 | 17.23 ± 3.43 | 26.53 ± 9.91 | 0.29 |
| RBC | 10^12^/L | 5.71 ± 2.72 | 2.96 ± 0.29 | 4.16 ± 1.45 | 7.51 ± 3.62 | 0.18 |
| HGB | g/L | 150.67 ± 4.51 | 154.67 ± 2.52 | 154 ± 2.65 | 150.67 ± 1.53 | 0.28 |
| HCT | % | 29.67 ± 14.17 | 14.97 ± 1.5 | 20.8 ± 6.36 | 38.13 ± 18.22 | 0.17 |
| MCV | fL | 52.07 ± 3.3 | 50.83 ± 1.59 | 50.53 ± 2.48 | 51 ± 0.78 | 0.85 |
| MCH | pg | 29.83 ± 11.12 | 52.57 ± 4.54 | 39.53 ± 11.12 | 25.4 ± 16.71 | 0.09 |
| MCHC | g/L | 577 ± 221.47 | 1039.67 ± 99.96 | 780 ± 196.15 | 497.67 ± 323.33 | 0.07 |
| RDW | % | 26.27 ± 8.57 | 37.87 ± 1.31 | 33.47 ± 6.95 | 24.1 ± 11.09 | 0.20 |
| PLT | 10^9^/L | 1318.67 ± 114.31 | 1816.67 ± 361.2 | 1468.33 ± 294.29 | 1272.67 ± 41.86 | 0.09 |
| MPV | fL | 6 ± 0.61 | 5.97 ± 0.21 | 5.87 ± 0.12 | 5.73 ± 0.21 | 0.78 |
| PDW |  | 16.97 ± 0.61 | 17.6 ± 0.44 | 16.97 ± 0.51 | 16.57 ± 0.4 | 0.16 |
